# Supplementary material for: Pan-cancer analysis reveals TAp63-regulated oncogenic lncRNAs that promote cancer progression through AKT activation
Source: Nat Commun. 2020 Oct 14;11:5156. doi: 10.1038/s41467-020-18973-w (PMC7561725; doi:10.1038/s41467-020-18973-w)
Supplement: Supplementary file 1 — Supplementary Information [file 41467_2020_18973_MOESM1_ESM.pdf]

SUPPLEMENTARY INFORMATION

Pan-cancer analysis reveals TAp63-regulated lncRNAs (TROLLs) that promote cancer progression through AKT activation

Napoli M., et al.

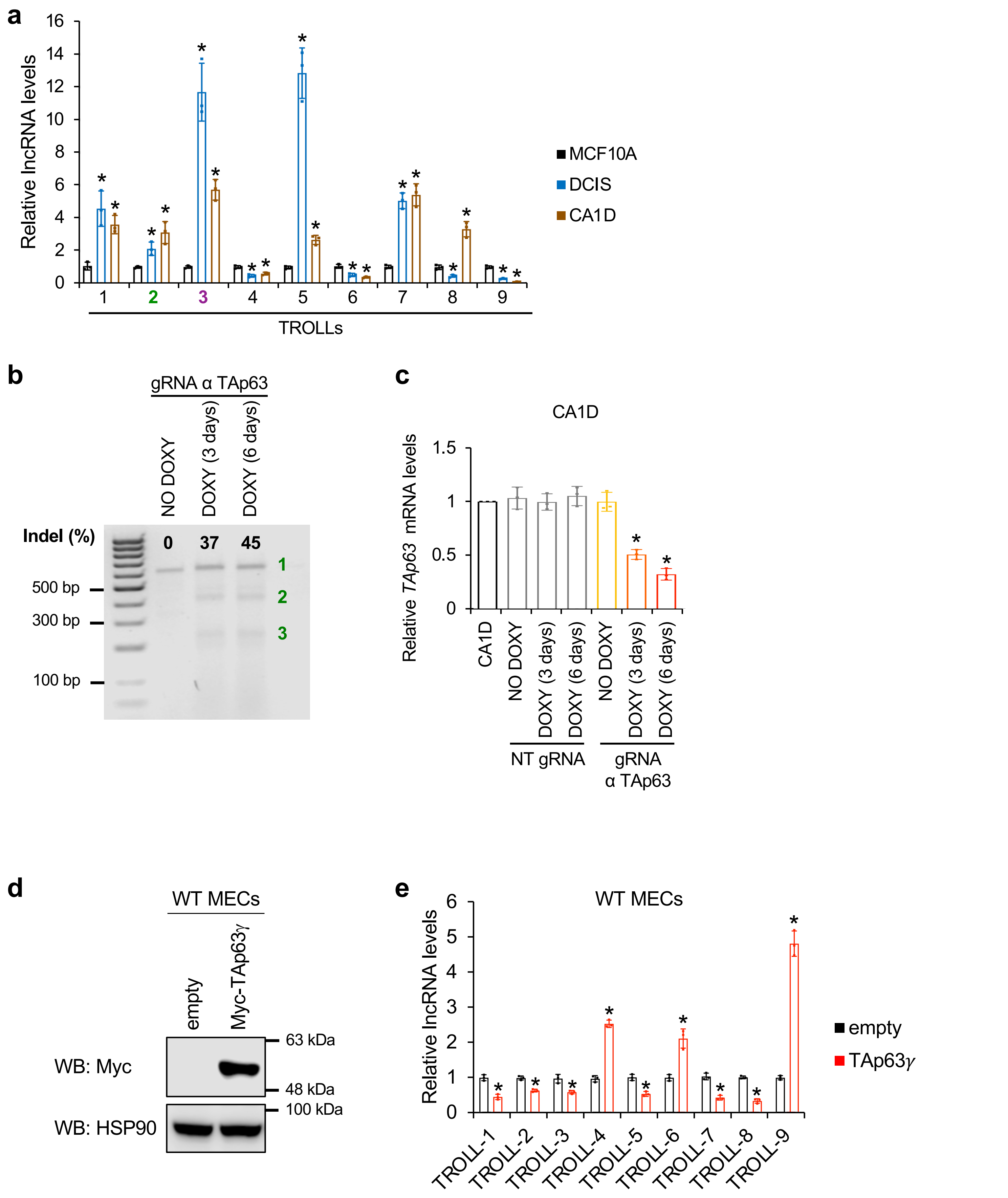

a
Relative lncRNA levels
MCF10A
DCIS
CA1D
TROLLs
b
gRNA α TAp63
NO DOXY
DOXY (3 days)
DOXY (6 days)
Indel (%)
0
37
45
500 bp
300 bp
100 bp
c
CA1D
Relative TAp63 mRNA levels
CA1D
NO DOXY
DOXY (3 days)
DOXY (6 days)
NT gRNA
gRNA α TAp63
d
WT MECs
empty
Myc-TAp63γ
WB: Myc
WB: HSP90
63 kDa
48 kDa
100 kDa
e
WT MECs
Relative lncRNA levels
empty
TAp63γ
TROLL-1
TROLL-2
TROLL-3
TROLL-4
TROLL-5
TROLL-6
TROLL-7
TROLL-8
TROLL-9

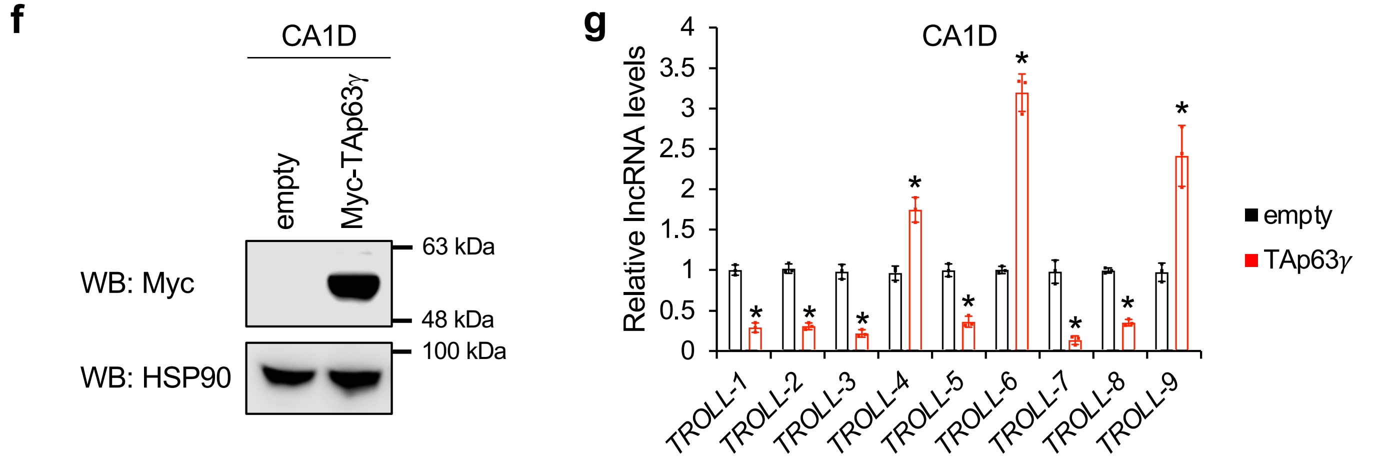

h

Element	Location	TAp63 Binding Site	MM / spacer
TROLL-1 promoter	-2213 to -2238	acaCATGctg tgtgcc aggCATGccc	2 / 6
TROLL-2 promoter	-4269 to -4240	aggCATGttc tagaaagaa agtCATGtac	2 / 9
TROLL-3 promoter	-3825 to -3800	aggCATGagc cacagc accCATGccc	4 / 6
TROLL-4 promoter	-4898 to -4919	gggCATGtca a aatCATGcac	3 / 1
TROLL-5 promoter	-2297 to -2320	ggaCATGcgc ctg tgcCATGccc	3 / 3
TROLL-6 promoter	-2264 to -2287	cggCATGacg tgt tttCATGact	6 / 3
TROLL-7 promoter	-3851 to -3874	cttCATGtct gac ctgCATGtct	5 / 3
TROLL-8 promoter	-2013 to -2034	gaaCATGatc g atcCATGtca	3 / 1
TROLL-9 promoter	-3254 to -3278	gggCATGgcc ttaa gcaCATGggc	4 / 4

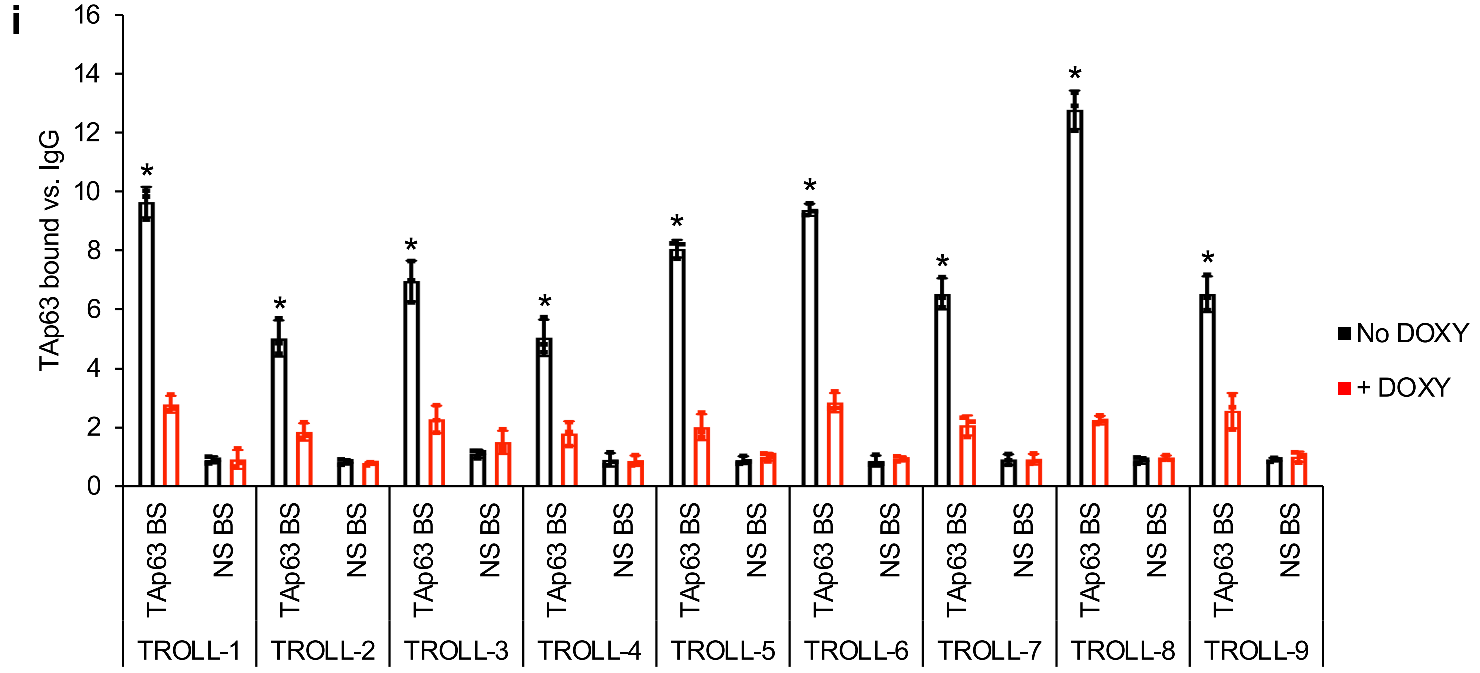

Supplementary Fig. 1 TAp63 regulates lncRNAs in breast cancer progression. **a** qRT-PCR of the 9 conserved lncRNAs differentially expressed in the MCF10A human breast cancer progression model. **b** Frequency of insertions/deletions occurring in the TAp63 locus in CA1D cells infected with *TAp63* targeting gRNA. **c** qRT-PCR for *TAp63* in CA1D cells treated as in (**b**). **d** Representative western blot of Myc-tag TAp63γ in WT Mammary Epithelial Cells (MECs) transfected with either pcDNA3 Myc-TAp63γ or pcDNA3, as a negative control. **e** qRT-PCR of the indicated lncRNAs in the same cells treated as in (**d**). **f** Representative WB of Myc-tag TAp63γ in CA1D cells transfected with either pcDNA3 Myc-TAp63γ or pcDNA3, as a negative control. **g** qRT-PCR of the indicated lncRNAs in the same cells treated as in (**f**). **h** Table listing the TAp63 binding sites on the promoters of the indicated lncRNAs. Number of mismatches to the TAp63 consensus binding site are shown in red text. MM indicates the number of mismatches to the TAp63 consensus binding site. Spacer indicates the number of nucleotides between two half sites. **i** qRT-PCR of TAp63 ChIP assays on the promoters of the indicated lncRNAs. CA1D cells infected with *TAp63* targeting gRNA were either left untreated (No doxy) or treated with doxycycline (+ doxy). Graphs represent the individual data points, mean ± SD of 3 independent experiments. P value (*$p < 0.05$) was calculated by two-tailed unpaired Student's t-test. Gels and blots are representative of $n = 3$ biological replicates. Source data are provided as Source data file.

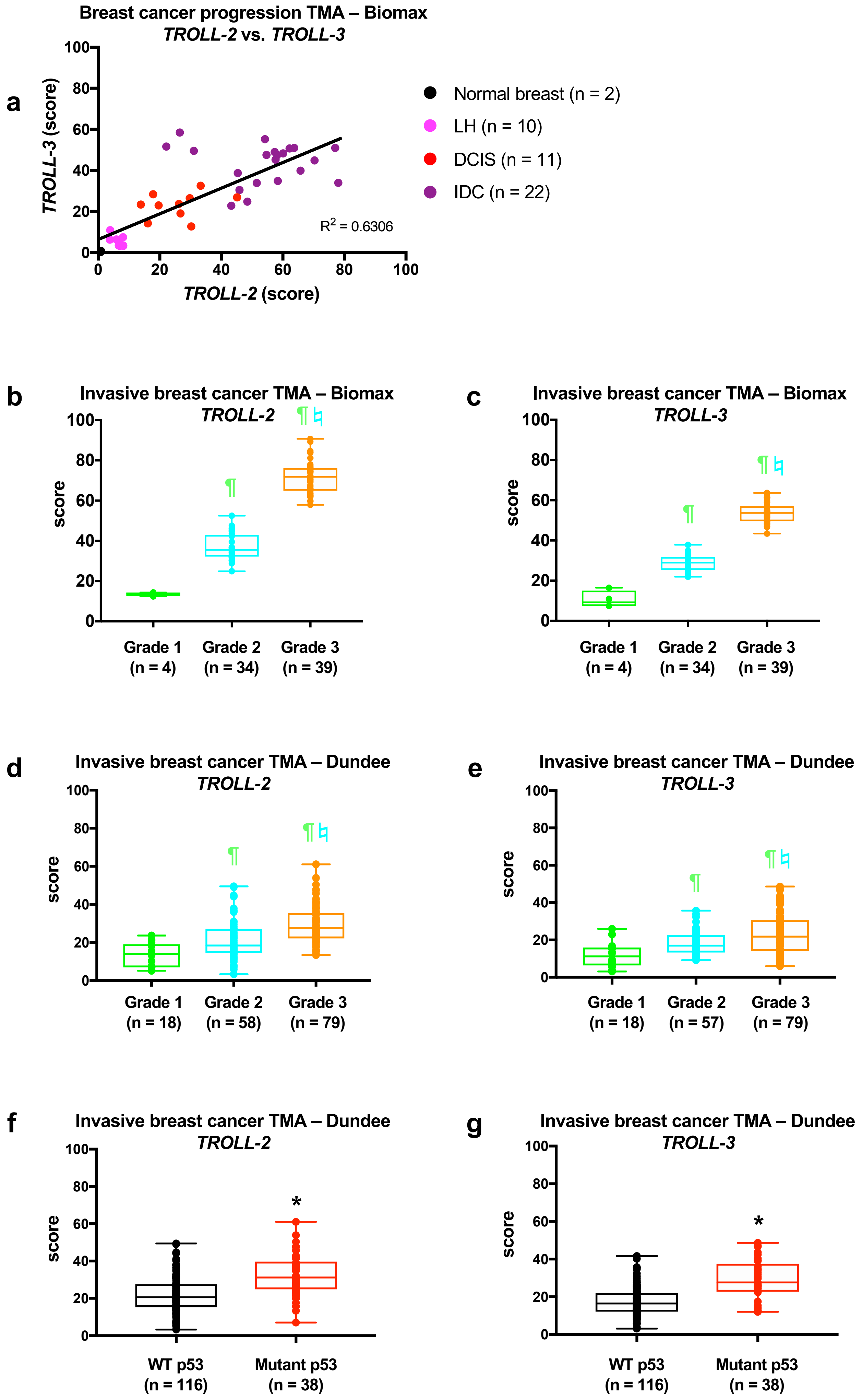

a
Breast cancer progression TMA – Biomax
TROLL-2 vs. TROLL-3
TROLL-3 (score)
TROLL-2 (score)
R^2 = 0.6306
Normal breast (n = 2)
LH (n = 10)
DCIS (n = 11)
IDC (n = 22)
b
Invasive breast cancer TMA – Biomax
TROLL-2
score
Grade 1 (n = 4)
Grade 2 (n = 34)
Grade 3 (n = 39)
c
Invasive breast cancer TMA – Biomax
TROLL-3
score
Grade 1 (n = 4)
Grade 2 (n = 34)
Grade 3 (n = 39)
d
Invasive breast cancer TMA – Dundee
TROLL-2
score
Grade 1 (n = 18)
Grade 2 (n = 58)
Grade 3 (n = 79)
e
Invasive breast cancer TMA – Dundee
TROLL-3
score
Grade 1 (n = 18)
Grade 2 (n = 57)
Grade 3 (n = 79)
f
Invasive breast cancer TMA – Dundee
TROLL-2
score
WT p53 (n = 116)
Mutant p53 (n = 38)
g
Invasive breast cancer TMA – Dundee
TROLL-3
score
WT p53 (n = 116)
Mutant p53 (n = 38)

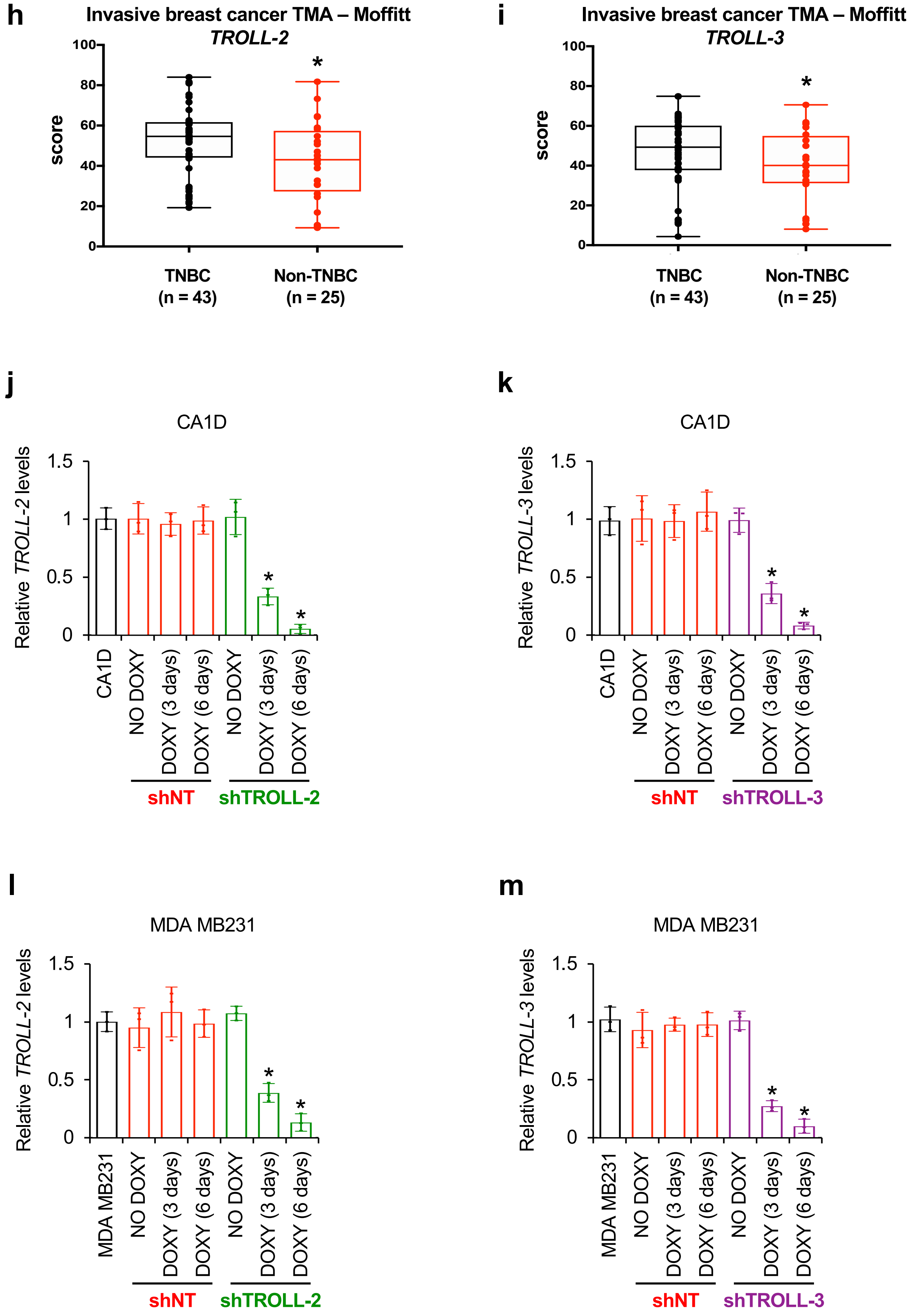

h
Invasive breast cancer TMA – Moffitt
TROLL-2
score
100
80
60
40
20
0
*
TNBC
(n = 43)
Non-TNBC
(n = 25)
i
Invasive breast cancer TMA – Moffitt
TROLL-3
score
100
80
60
40
20
0
*
TNBC
(n = 43)
Non-TNBC
(n = 25)
j
CA1D
Relative TROLL-2 levels
1.5
1
0.5
0
*
*
CA1D
NO DOXY
DOXY (3 days)
DOXY (6 days)
NO DOXY
DOXY (3 days)
DOXY (6 days)
shNT
shTROLL-2
k
CA1D
Relative TROLL-3 levels
1.5
1
0.5
0
*
*
CA1D
NO DOXY
DOXY (3 days)
DOXY (6 days)
NO DOXY
DOXY (3 days)
DOXY (6 days)
shNT
shTROLL-3
l
MDA MB231
Relative TROLL-2 levels
1.5
1
0.5
0
*
*
MDA MB231
NO DOXY
DOXY (3 days)
DOXY (6 days)
NO DOXY
DOXY (3 days)
DOXY (6 days)
shNT
shTROLL-2
m
MDA MB231
Relative TROLL-3 levels
1.5
1
0.5
0
*
*
MDA MB231
NO DOXY
DOXY (3 days)
DOXY (6 days)
NO DOXY
DOXY (3 days)
DOXY (6 days)
shNT
shTROLL-3

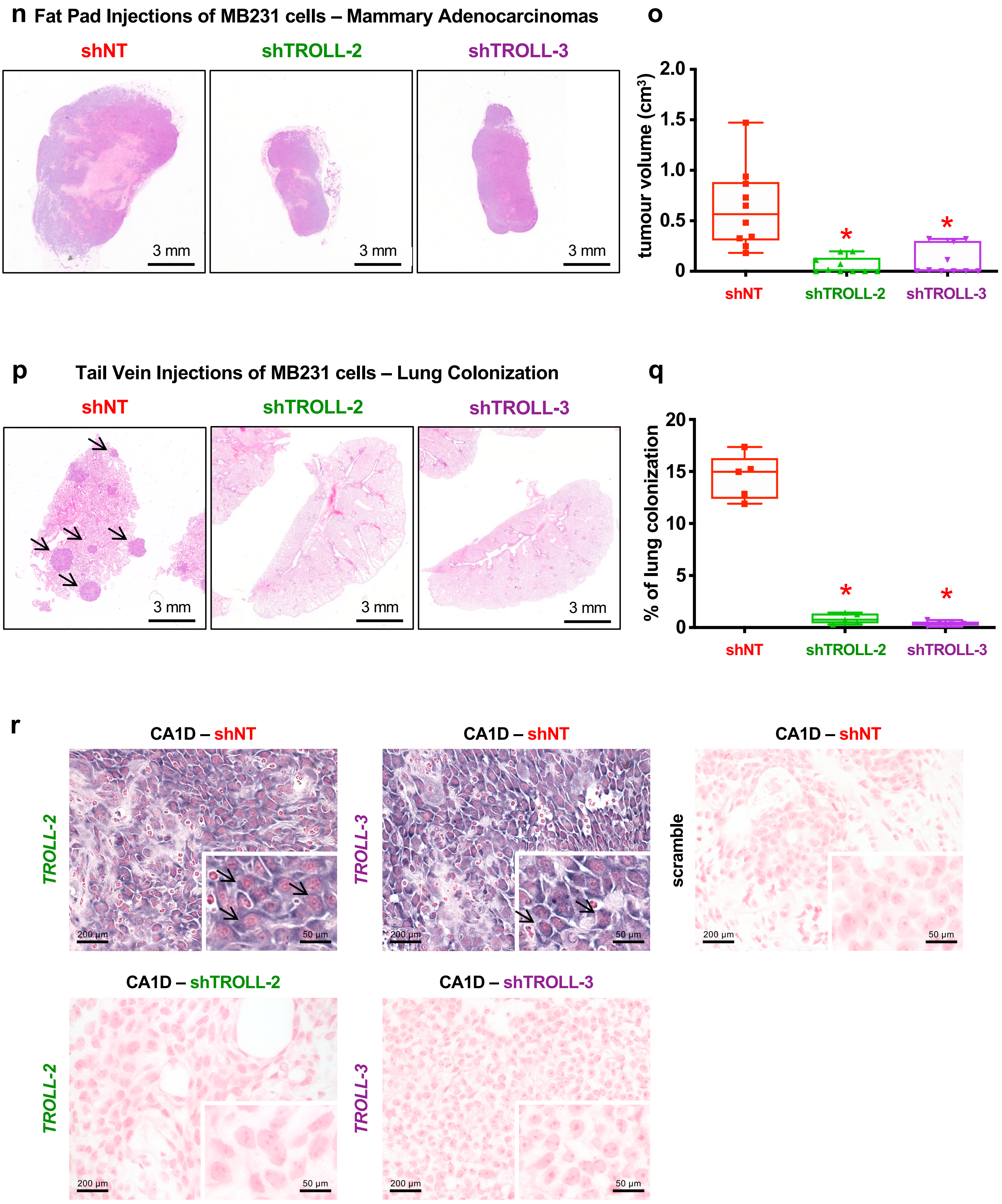
n Fat Pad Injections of MB231 cells – Mammary Adenocarcinomas
shNT
shTROLL-2
shTROLL-3
3 mm
o
tumour volume (cm³)
2.0
1.5
1.0
0.5
0
shNT
shTROLL-2
shTROLL-3
p Tail Vein Injections of MB231 cells – Lung Colonization
q
% of lung colonization
20
15
10
5
r
CA1D – shNT
TROLL-2
TROLL-3
scramble
CA1D – shTROLL-2
CA1D – shTROLL-3
200 µm
50 µm

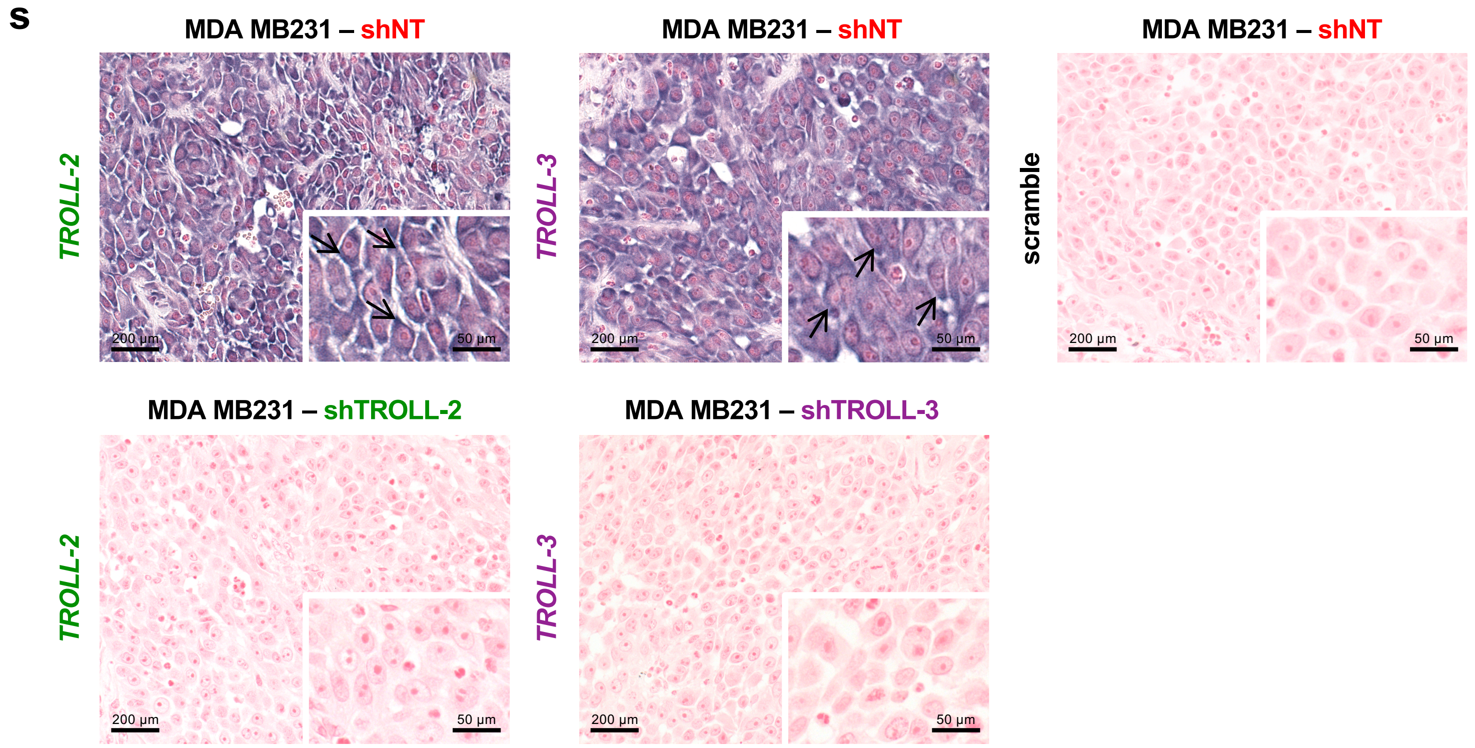

Supplementary Fig. 2 *TROLL-2* and *TROLL-3* are markers of breast cancer progression. **a** Correlation of the ISH scores of *TROLL-2* and *TROLL-3* shown in Fig. 2a,b. **b-e** Correlation of the ISH scores of *TROLL-2* and *TROLL-3* with tumour grade in the indicated TMAs. Data were analysed with two-way ANOVA. ¶ vs. grade 1, $P < 0.005$. ♮ vs. grade 2, $P < 0.005$. **f**,**g** Correlation of the ISH scores of *TROLL-2* (**f**) and *TROLL-3* (**g**) with *TP53* status in the indicated TMA. * vs. WT p53, $P < 0.005$, Welchs Student's *t* test. **h**,**i** Correlation of the ISH scores of *TROLL-2* (**h**) and *TROLL-3* (**i**) with TNBC vs. non-TNBC cases in the indicated TMA. * vs. TNBC, $P < 0.005$, Welchs *t* test. **j-m** qRT-PCR for *TROLL-2* and *TROLL-3* in either CA1D (**j**,**k**) or MDA MB-231 cells (**l**,**m**) infected with the indicated shRNAs. * vs. shNT no doxy, $P < 0.005$, two-tailed Student's *t* test. **n** Representative H&E stained cross sections of mammary adenocarcinomas derived from MDA MB-231 cells infected with the indicated shRNAs. **o** Tumour volume quantification of the tumours described in (**n**). $n = 10$ tumours, * vs. shNT, $P < 0.005$, two-tailed Student's *t* test. **p** Representative H&E stained cross sections of lung colonies derived from MDA MB-231 cells infected with the indicated shRNAs. **q** Quantification of the lung colonies described in (**p**). $n = 5$ mice for all groups, * vs. NT, $P < 0.005$, two-tailed Student's t test. **r**,**s** Representative images of ISH for *TROLL-2* (left panels) and *TROLL-3* (middle panels) in tumours derived from CA1D (**r**) or MDA MB-231 (**s**) cells. Micrographs are representative of $n = 10$ tumours. Graphs represent the individual data points, mean ± SD of 3 independent experiments. All boxplots represent the individual data points, median and whiskers (min to max method). Source data are provided as Source data file.

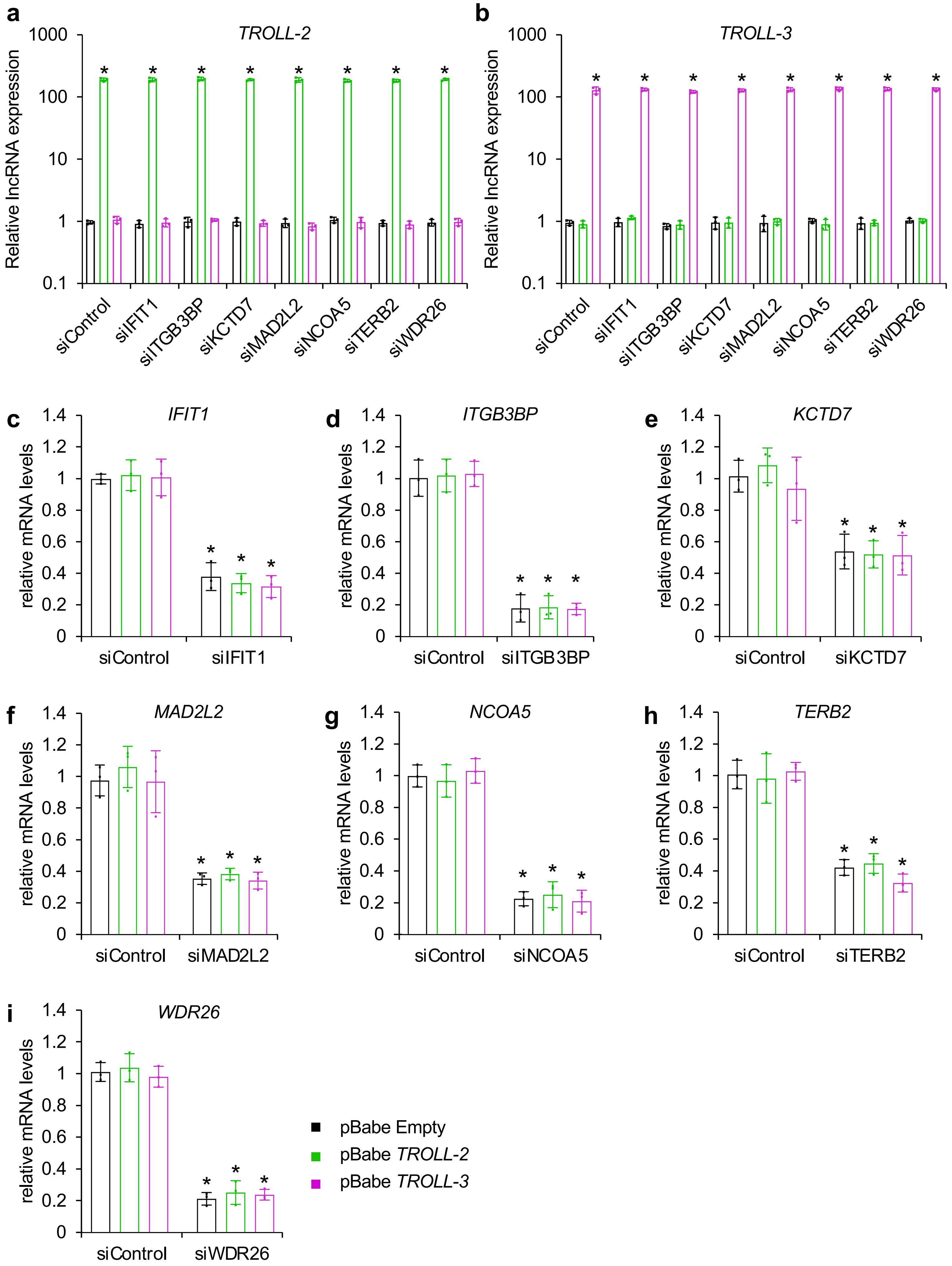

a
TROLL-2
Relative lncRNA expression
1000
100
10
1
0.1
siControl
siIFIT1
siITGB3BP
siKCTD7
siMAD2L2
siNCOA5
siTERB2
siWDR26
b
TROLL-3
c
IFIT1
relative mRNA levels
siIFIT1
d
ITGB3BP
siITGB3BP
e
KCTD7
siKCTD7
f
MAD2L2
siMAD2L2
g
NCOA5
siNCOA5
h
TERB2
siTERB2
i
WDR26
siWDR26
pBabe Empty
pBabe TROLL-2
pBabe TROLL-3

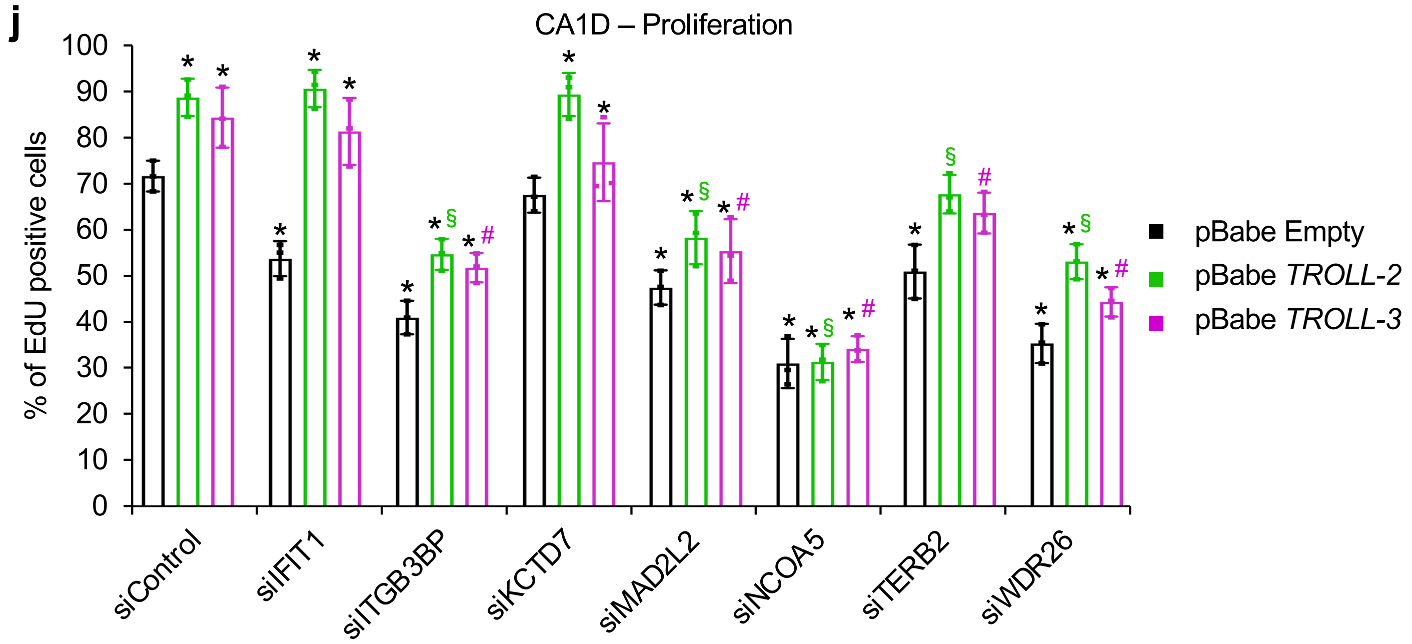

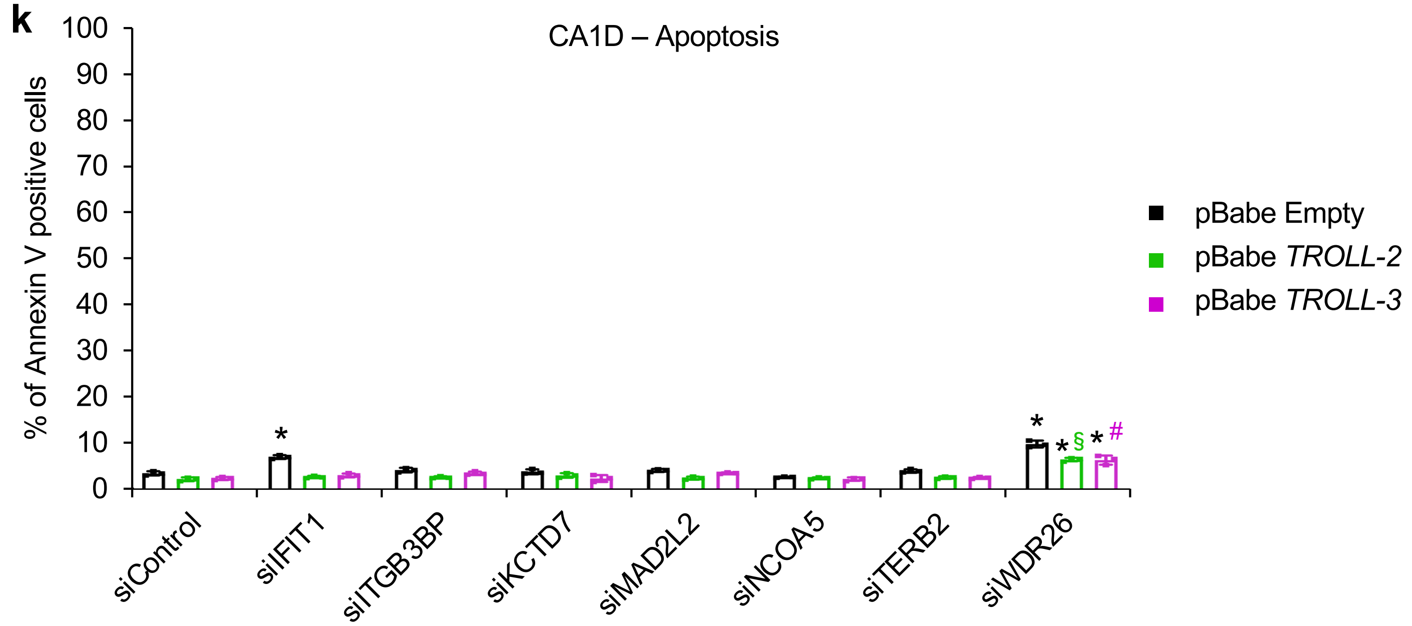

Supplementary Fig. 3 *TROLL-2* and *TROLL-3* promote cancer progression via WDR26. **a-i** qRT-PCR for *TROLL-2* (**a**), *TROLL-3* (**b**), *IFIT1* (**c**), *ITGB3BP* (**d**), *KCDT7* (**e**), *MAD2L2* (**f**), *NCOA5* (**g**), *TERB2* (**h**), and *WDR26* (**i**) in CA1D cells overexpressing either *TROLL-2*, *TROLL-3*, or the empty vector as a negative control, and transfected with the indicated siRNAs. Graphs represent the individual data points, mean ± SD and analysed with two-tailed Student's *t* test, $n = 3$ biological replicates, * vs. pBabe Empty siControl, $P < 0.005$. **j**,**k** Quantification for EdU (**j**) and annexin V (**k**) of CA1D cells treated as in **a-i**. Graphs represent the individual data points, mean ± SD and analysed with two-tailed Student's *t* test, $n = 3$ biological replicates, * vs. pBabe Empty siControl, $P < 0.005$. § vs. pBabe *TROLL-2* siControl, $P < 0.005$. # vs. pBabe *TROLL-3* siControl, $P < 0.005$. Source data are provided as Source data file.

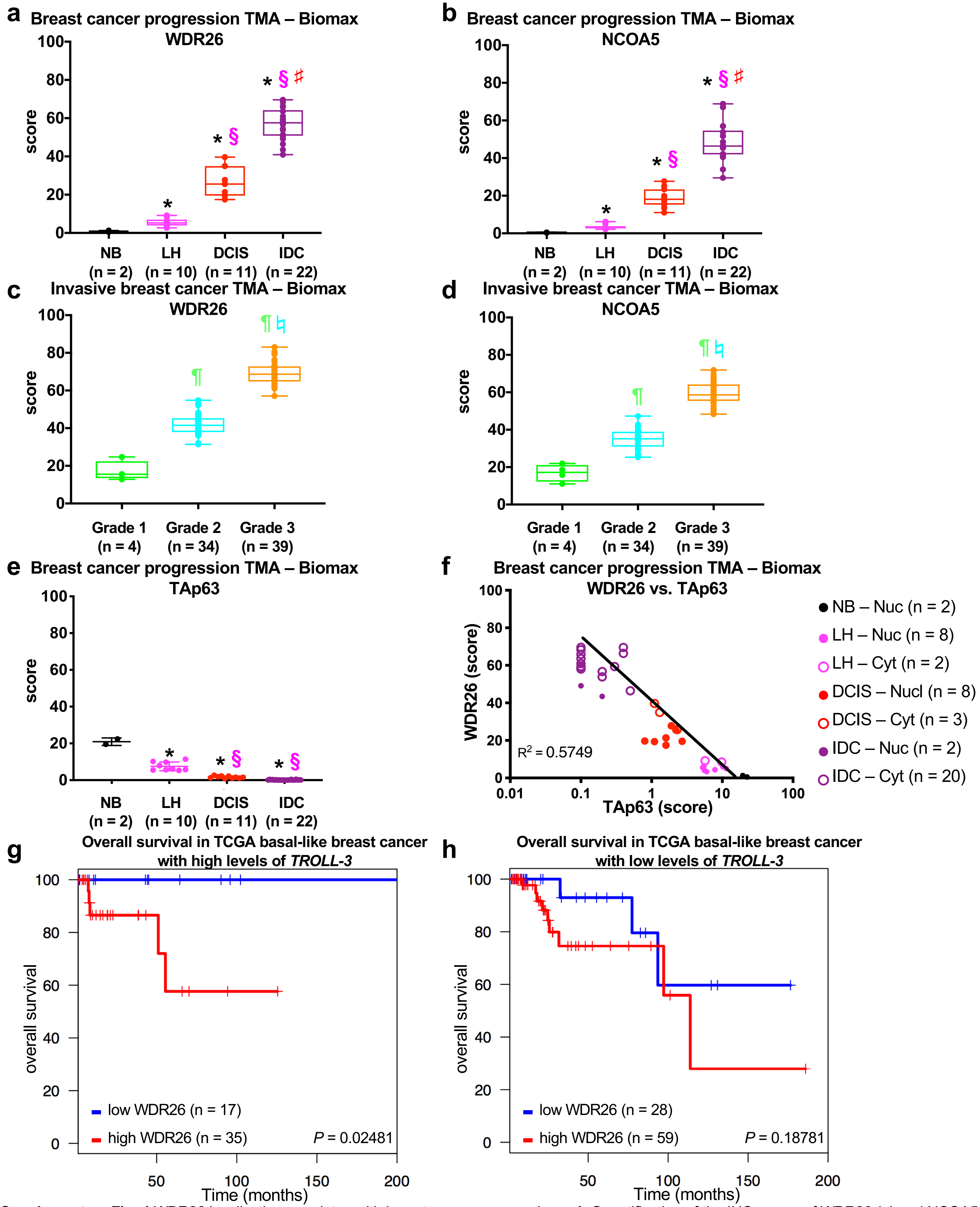

Supplementary Fig. 4 WDR26 localization correlates with breast cancer progression. **a**,**b** Quantification of the IHC scores of WDR26 (**a**) and NCOA5 (**b**) in the indicated TMAs. **c**,**d** Quantification of the IHC scores of WDR26 (**c**) and NCOA5 (**d**) in the indicated TMAs. **e** Quantification of the IHC scores of TAp63 in the indicated TMA. **f** Correlation of the IHC score and cellular distribution of WDR26 with the IHC score of TAp63 in the same TMA as in (**e**). **g**,**h** Kaplan-Meier curves of overall TCGA breast cancer survival based on the levels of *TROLL-3*. COXPH *P* values are shown. In a-e, boxplots represent the individual data points, median and whiskers (min to max method). Data were analysed with two-way ANOVA. * vs. normal breast tissue, § vs. lobular hyperplasia, # vs. ductal carcinoma in situ, ¶ vs. grade 1, ♮ vs. grade 2, $P < 0.005$. Source data are provided as Source data file.

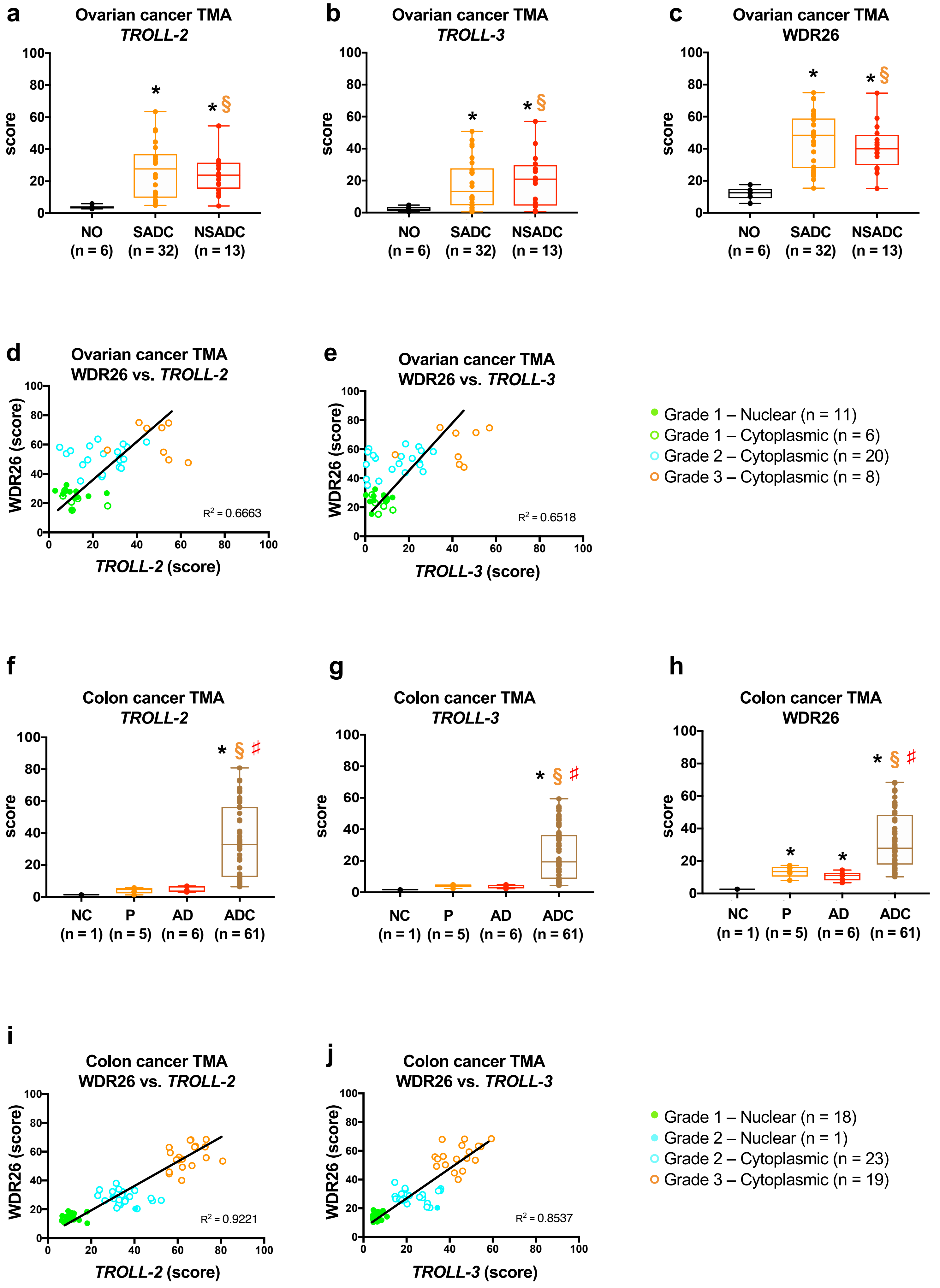

a
Ovarian cancer TMA
TROLL-2
b
Ovarian cancer TMA
TROLL-3
c
Ovarian cancer TMA
WDR26
score
NO (n = 6)
SADC (n = 32)
NSADC (n = 13)
d
Ovarian cancer TMA
WDR26 vs. TROLL-2
WDR26 (score)
TROLL-2 (score)
R2 = 0.6663
e
Ovarian cancer TMA
WDR26 vs. TROLL-3
WDR26 (score)
TROLL-3 (score)
R2 = 0.6518
Grade 1 – Nuclear (n = 11)
Grade 1 – Cytoplasmic (n = 6)
Grade 2 – Cytoplasmic (n = 20)
Grade 3 – Cytoplasmic (n = 8)
f
Colon cancer TMA
TROLL-2
g
Colon cancer TMA
TROLL-3
h
Colon cancer TMA
WDR26
score
NC (n = 1)
P (n = 5)
AD (n = 6)
ADC (n = 61)
i
Colon cancer TMA
WDR26 vs. TROLL-2
WDR26 (score)
TROLL-2 (score)
R2 = 0.9221
j
Colon cancer TMA
WDR26 vs. TROLL-3
WDR26 (score)
TROLL-3 (score)
R2 = 0.8537
Grade 1 – Nuclear (n = 18)
Grade 2 – Nuclear (n = 1)
Grade 2 – Cytoplasmic (n = 23)
Grade 3 – Cytoplasmic (n = 19)

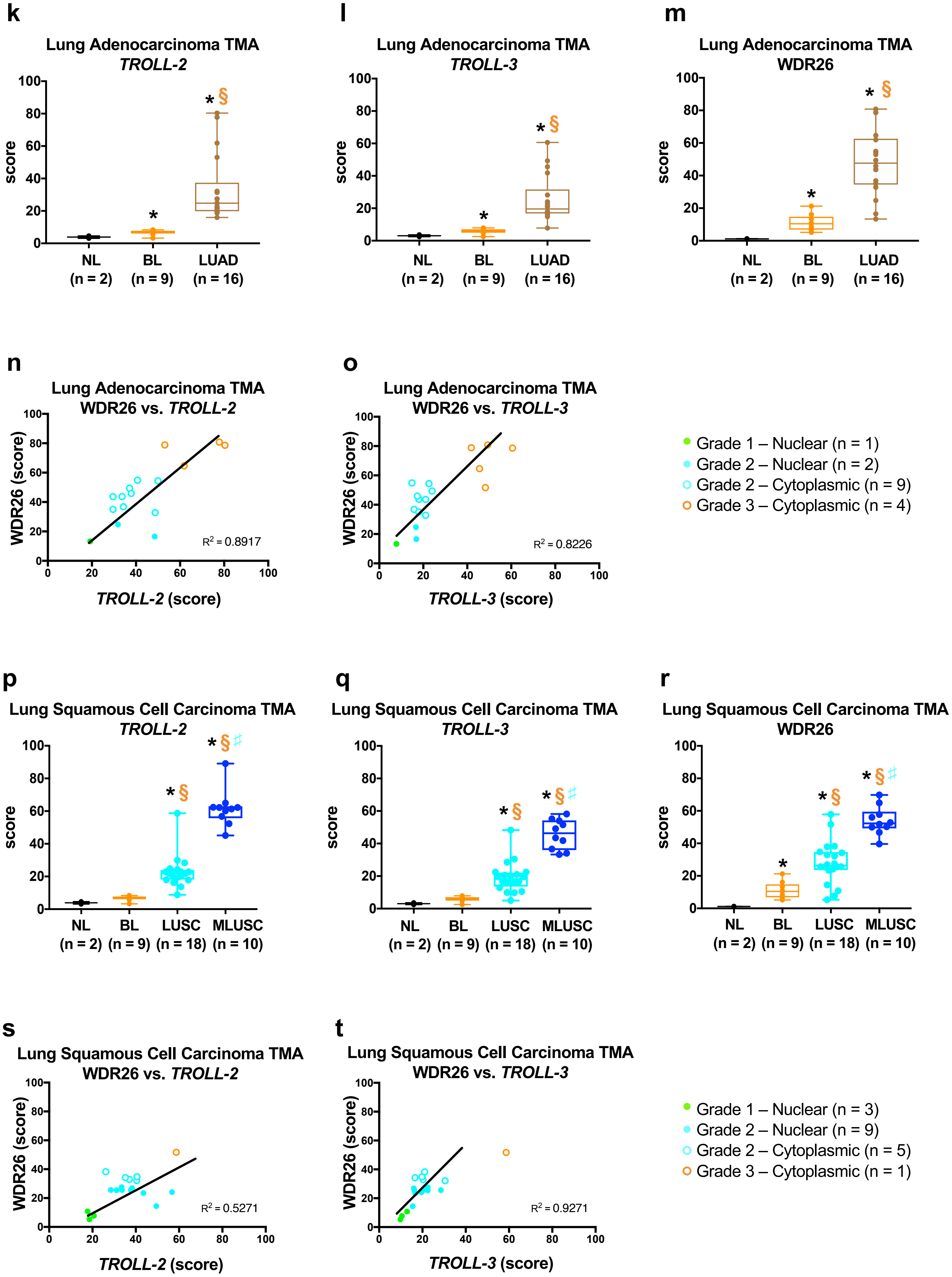
k
Lung Adenocarcinoma TMA
TROLL-2
l
Lung Adenocarcinoma TMA
TROLL-3
m
Lung Adenocarcinoma TMA
WDR26
score
NL (n = 2)
BL (n = 9)
LUAD (n = 16)
n
Lung Adenocarcinoma TMA
WDR26 vs. TROLL-2
WDR26 (score)
TROLL-2 (score)
R2 = 0.8917
o
Lung Adenocarcinoma TMA
WDR26 vs. TROLL-3
TROLL-3 (score)
R2 = 0.8226
Grade 1 – Nuclear (n = 1)
Grade 2 – Nuclear (n = 2)
Grade 2 – Cytoplasmic (n = 9)
Grade 3 – Cytoplasmic (n = 4)
p
Lung Squamous Cell Carcinoma TMA
TROLL-2
q
Lung Squamous Cell Carcinoma TMA
TROLL-3
r
Lung Squamous Cell Carcinoma TMA
WDR26
NL (n = 2)
BL (n = 9)
LUSC (n = 18)
MLUSC (n = 10)
s
Lung Squamous Cell Carcinoma TMA
WDR26 vs. TROLL-2
R2 = 0.5271
t
Lung Squamous Cell Carcinoma TMA
WDR26 vs. TROLL-3
R2 = 0.9271
Grade 1 – Nuclear (n = 3)
Grade 2 – Nuclear (n = 9)
Grade 2 – Cytoplasmic (n = 5)
Grade 3 – Cytoplasmic (n = 1)

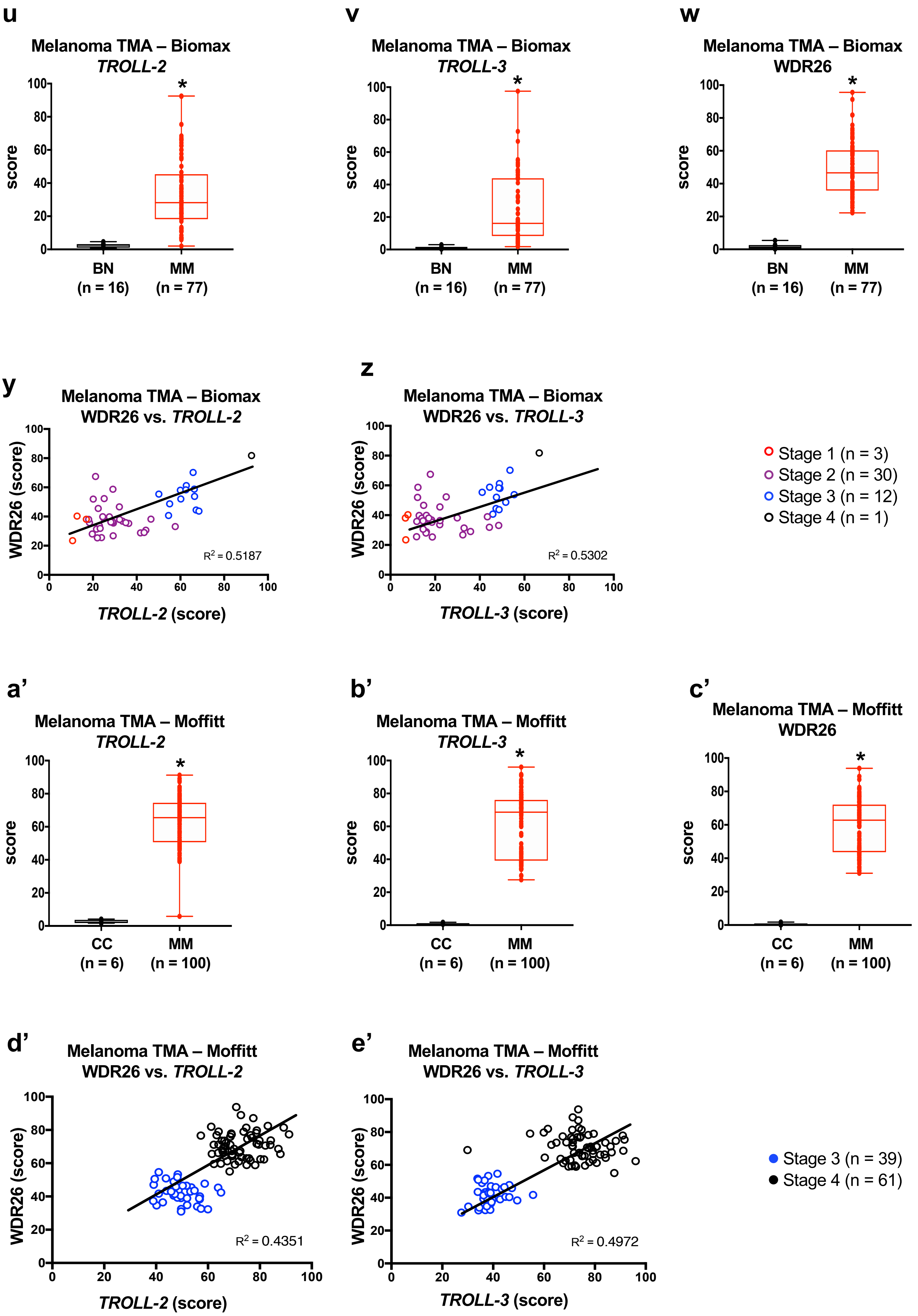

u
Melanoma TMA – Biomax
TROLL-2
score
BN (n = 16)
MM (n = 77)
v
Melanoma TMA – Biomax
TROLL-3
score
BN (n = 16)
MM (n = 77)
w
Melanoma TMA – Biomax
WDR26
score
BN (n = 16)
MM (n = 77)
y
Melanoma TMA – Biomax
WDR26 vs. TROLL-2
WDR26 (score)
TROLL-2 (score)
R2 = 0.5187
z
Melanoma TMA – Biomax
WDR26 vs. TROLL-3
WDR26 (score)
TROLL-3 (score)
R2 = 0.5302
Stage 1 (n = 3)
Stage 2 (n = 30)
Stage 3 (n = 12)
Stage 4 (n = 1)
a'
Melanoma TMA – Moffitt
TROLL-2
score
CC (n = 6)
MM (n = 100)
b'
Melanoma TMA – Moffitt
TROLL-3
score
CC (n = 6)
MM (n = 100)
c'
Melanoma TMA – Moffitt
WDR26
score
CC (n = 6)
MM (n = 100)
d'
Melanoma TMA – Moffitt
WDR26 vs. TROLL-2
WDR26 (score)
TROLL-2 (score)
R2 = 0.4351
e'
Melanoma TMA – Moffitt
WDR26 vs. TROLL-3
WDR26 (score)
TROLL-3 (score)
R2 = 0.4972
Stage 3 (n = 39)
Stage 4 (n = 61)

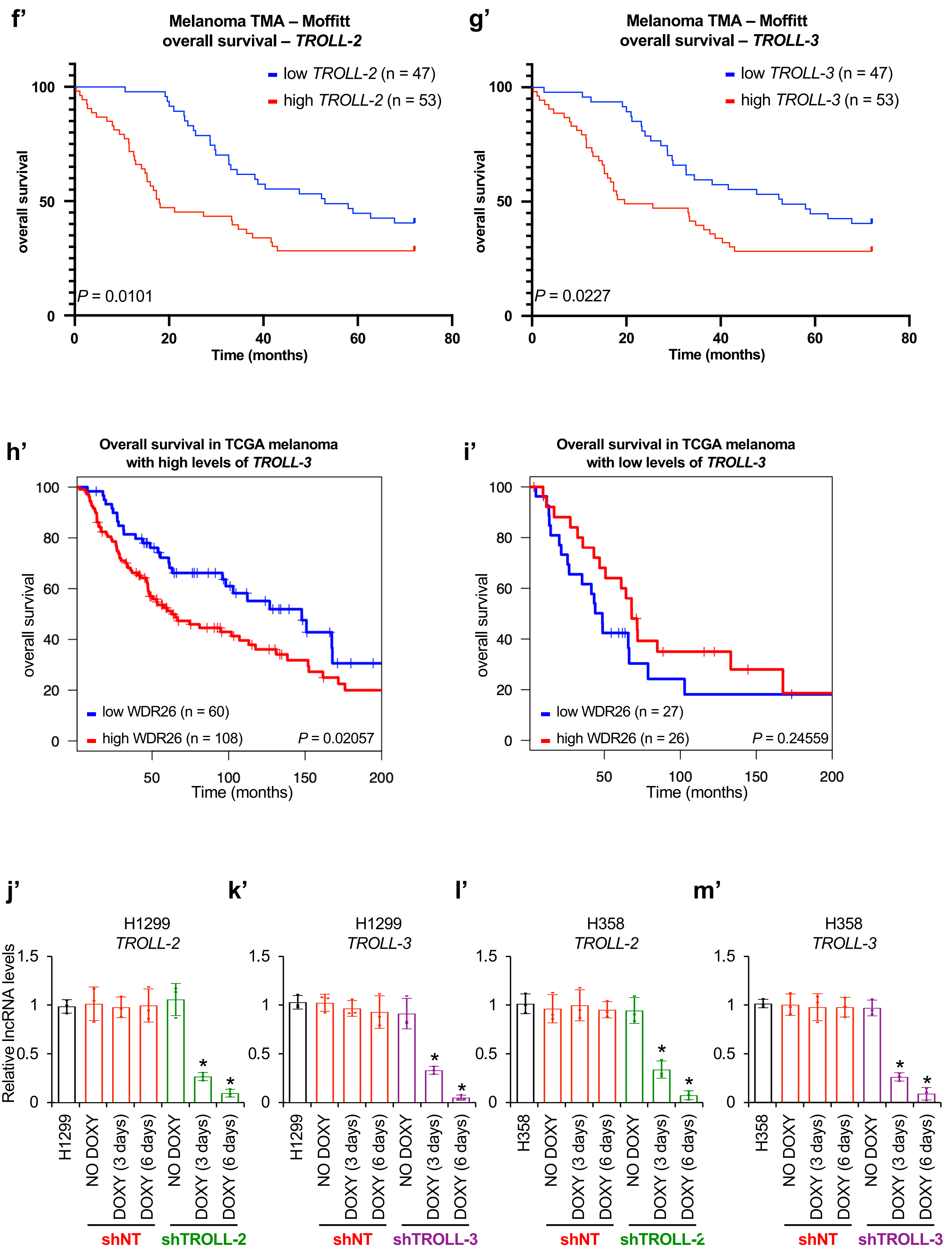
f’
Melanoma TMA – Moffitt
overall survival – TROLL-2
low TROLL-2 (n = 47)
high TROLL-2 (n = 53)
overall survival
P = 0.0101
Time (months)
g’
Melanoma TMA – Moffitt
overall survival – TROLL-3
low TROLL-3 (n = 47)
high TROLL-3 (n = 53)
overall survival
P = 0.0227
Time (months)
h’
Overall survival in TCGA melanoma
with high levels of TROLL-3
overall survival
low WDR26 (n = 60)
high WDR26 (n = 108)
P = 0.02057
Time (months)
i’
Overall survival in TCGA melanoma
with low levels of TROLL-3
overall survival
low WDR26 (n = 27)
high WDR26 (n = 26)
P = 0.24559
Time (months)
j’
H1299
TROLL-2
Relative lncRNA levels
H1299
NO DOXY
DOXY (3 days)
DOXY (6 days)
NO DOXY
DOXY (3 days)
DOXY (6 days)
shNT
shTROLL-2
k’
H1299
TROLL-3
H1299
NO DOXY
DOXY (3 days)
DOXY (6 days)
NO DOXY
DOXY (3 days)
DOXY (6 days)
shNT
shTROLL-3
l’
H358
TROLL-2
H358
NO DOXY
DOXY (3 days)
DOXY (6 days)
NO DOXY
DOXY (3 days)
DOXY (6 days)
shNT
shTROLL-2
m’
H358
TROLL-3
H358
NO DOXY
DOXY (3 days)
DOXY (6 days)
NO DOXY
DOXY (3 days)
DOXY (6 days)
shNT
shTROLL-3

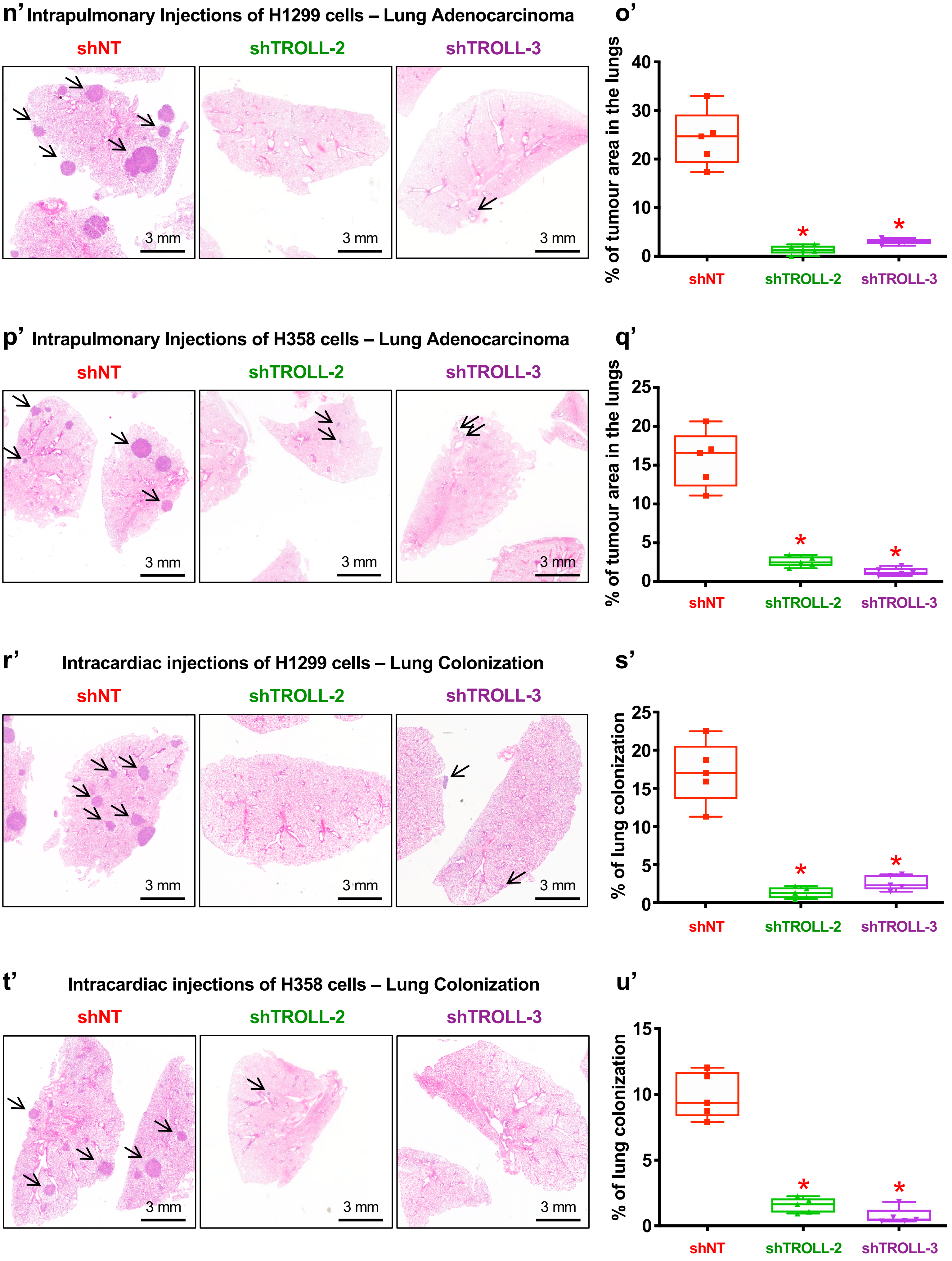
n' Intrapulmonary Injections of H1299 cells – Lung Adenocarcinoma
shNT
shTROLL-2
shTROLL-3
3 mm
3 mm
3 mm
o'
% of tumour area in the lungs
40
30
20
10
0
*
*
shNT
shTROLL-2
shTROLL-3
p' Intrapulmonary Injections of H358 cells – Lung Adenocarcinoma
shNT
shTROLL-2
shTROLL-3
3 mm
3 mm
3 mm
q'
% of tumour area in the lungs
25
20
15
10
5
0
*
*
shNT
shTROLL-2
shTROLL-3
r' Intracardiac injections of H1299 cells – Lung Colonization
shNT
shTROLL-2
shTROLL-3
3 mm
3 mm
3 mm
s'
% of lung colonization
25
20
15
10
5
0
*
*
shNT
shTROLL-2
shTROLL-3
t' Intracardiac injections of H358 cells – Lung Colonization
shNT
shTROLL-2
shTROLL-3
3 mm
3 mm
3 mm
u'
% of lung colonization
15
10
5
0
*
*
shNT
shTROLL-2
shTROLL-3

v’

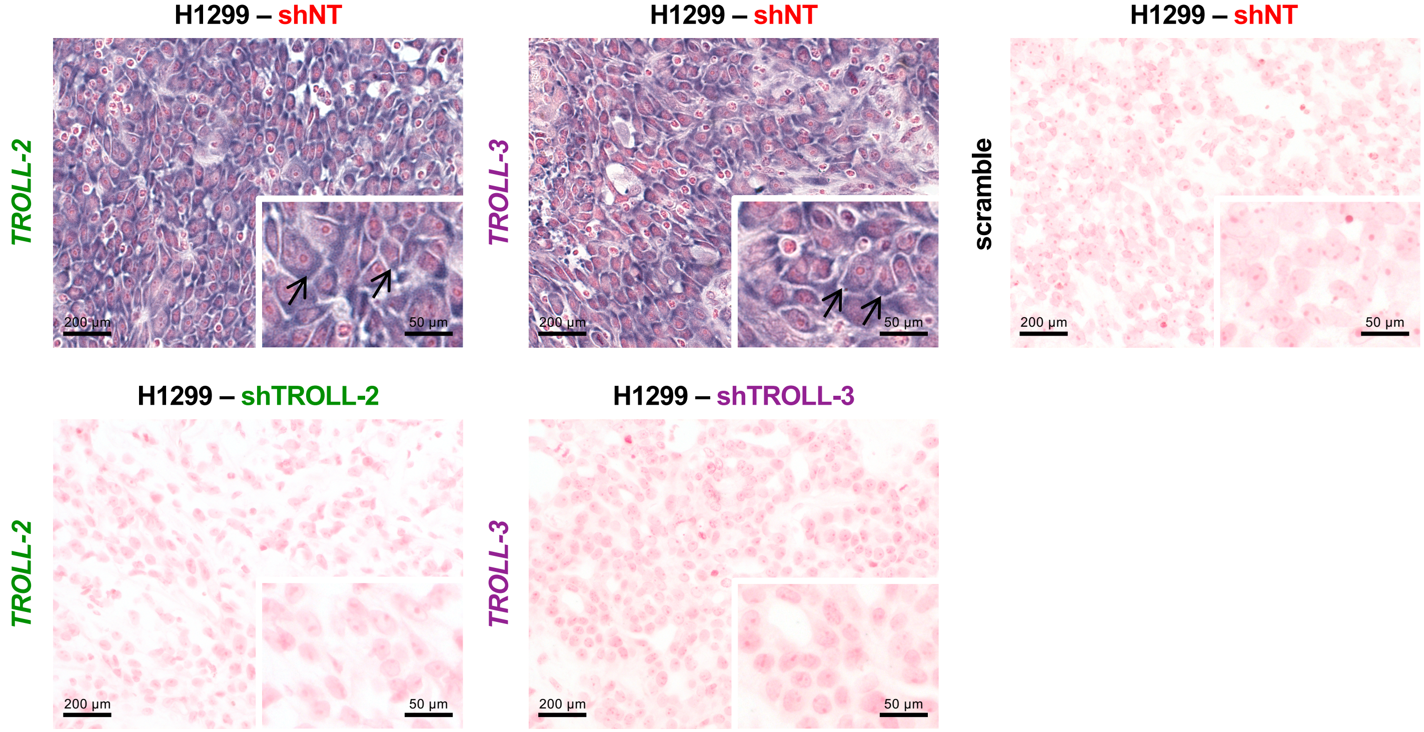
H1299 – shNT
H1299 – shNT
H1299 – shNT
TROLL-2
TROLL-3
scramble
200 µm
50 µm
H1299 – shTROLL-2
H1299 – shTROLL-3
TROLL-2
TROLL-3

w’

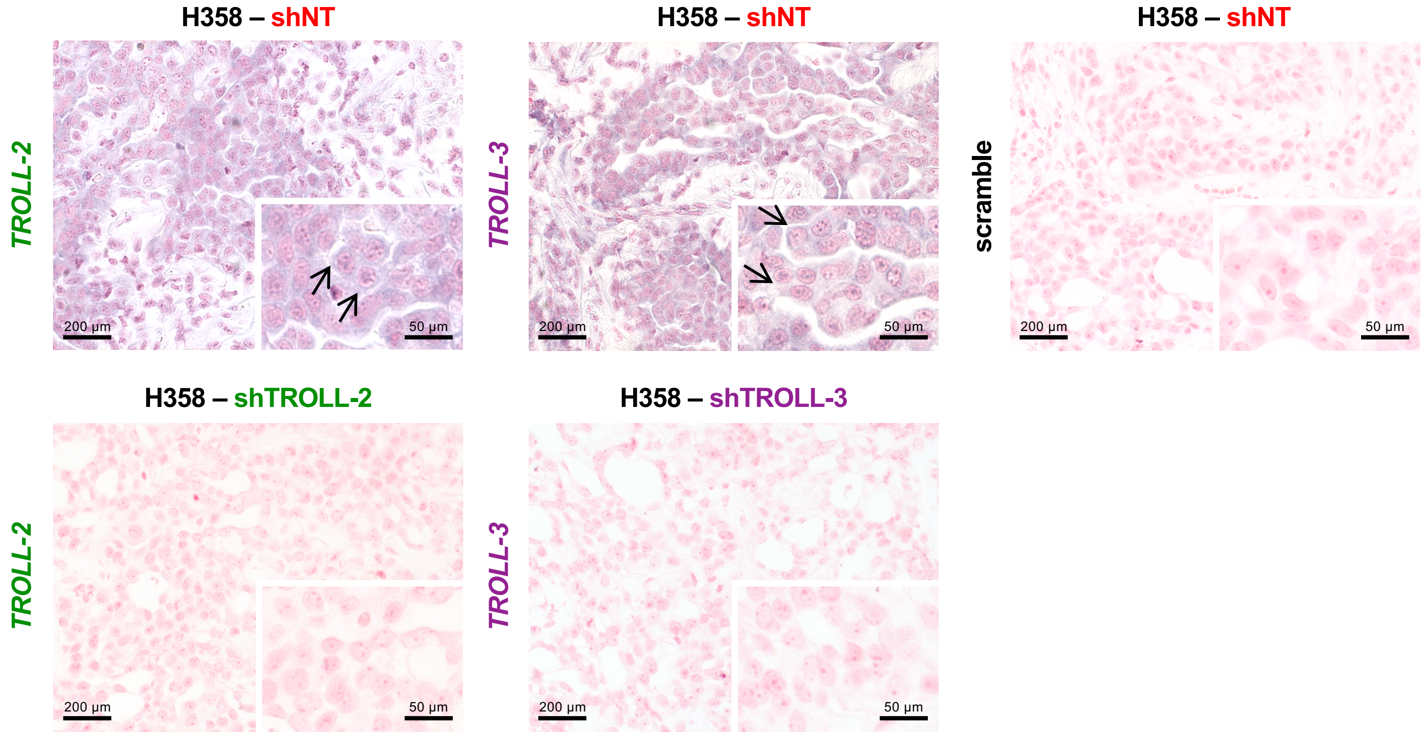
H358 – shNT
H358 – shNT
H358 – shNT
TROLL-2
TROLL-3
scramble
200 µm
50 µm
H358 – shTROLL-2
H358 – shTROLL-3
TROLL-2
TROLL-3

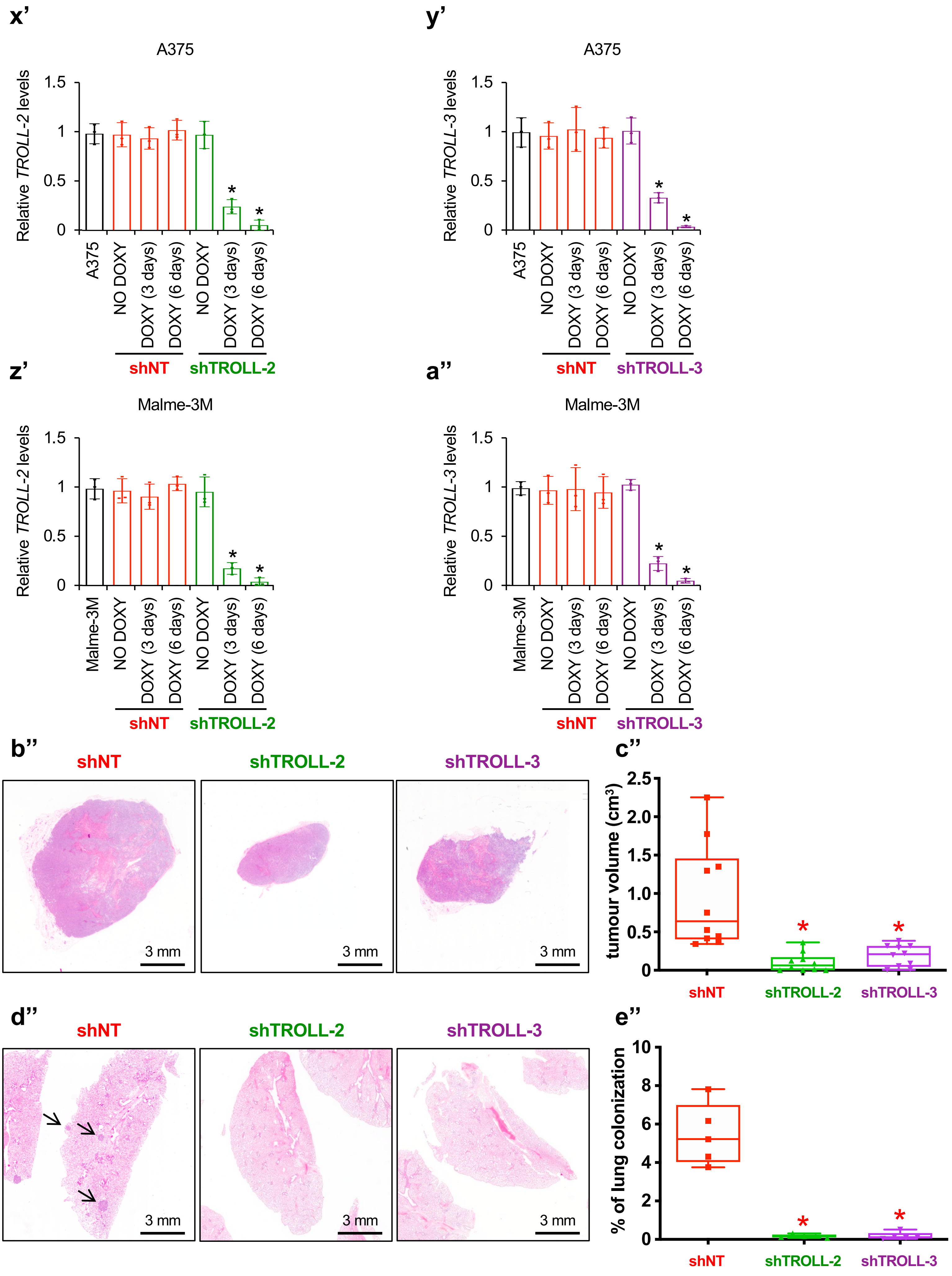

x'
A375
Relative TROLL-2 levels
1.5
1
0.5
0
A375
NO DOXY
DOXY (3 days)
DOXY (6 days)
NO DOXY
DOXY (3 days)
DOXY (6 days)
shNT
shTROLL-2
y'
A375
Relative TROLL-3 levels
shNT
shTROLL-3
z'
Malme-3M
Relative TROLL-2 levels
Malme-3M
shNT
shTROLL-2
a''
Malme-3M
Relative TROLL-3 levels
shNT
shTROLL-3
b''
shNT
shTROLL-2
shTROLL-3
3 mm
c''
tumour volume (cm³)
2.5
2.0
1.5
1.0
0.5
0
shNT
shTROLL-2
shTROLL-3
d''
shNT
shTROLL-2
shTROLL-3
3 mm
e''
% of lung colonization
10
8
6
4
2
0
shNT
shTROLL-2
shTROLL-3

f’’

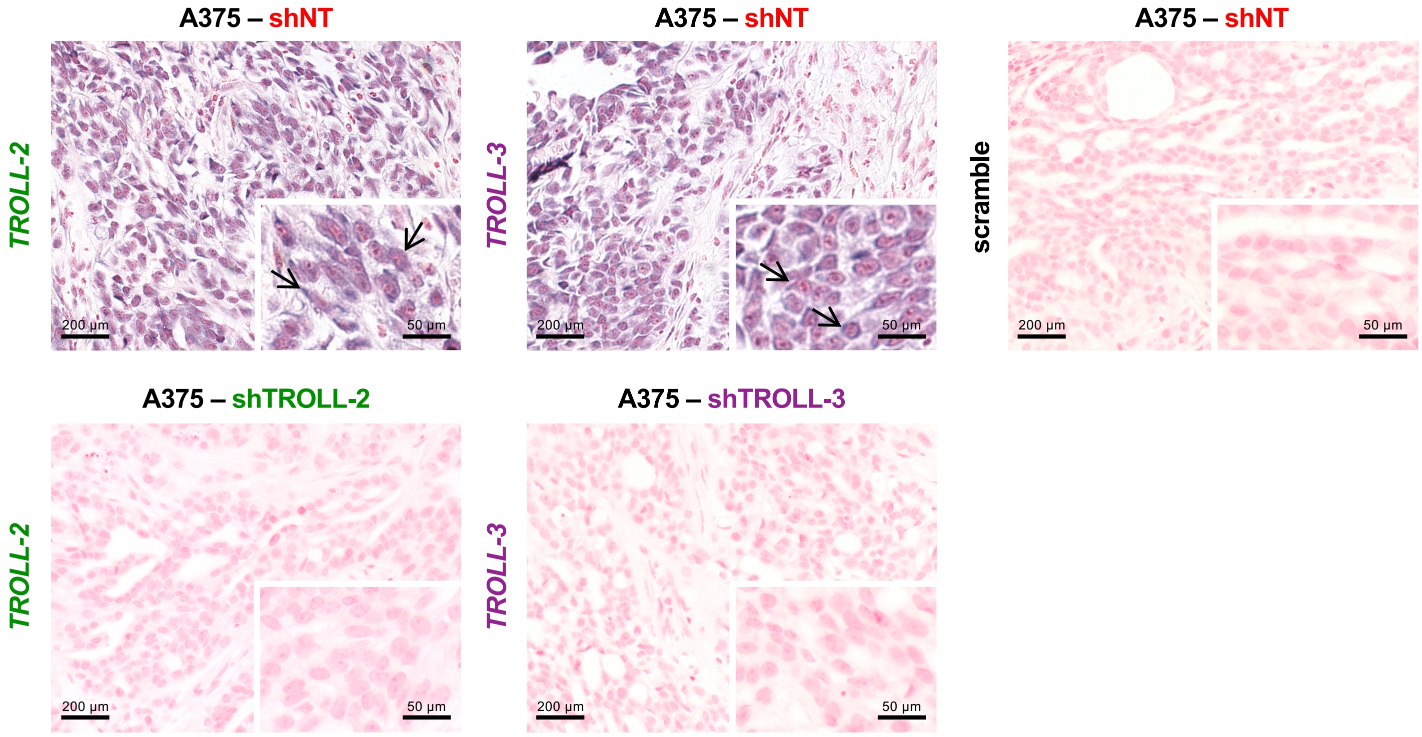
A375 – shNT
A375 – shNT
A375 – shNT
TROLL-2
TROLL-3
scramble
200 µm
50 µm
A375 – shTROLL-2
A375 – shTROLL-3
TROLL-2
TROLL-3

g’’

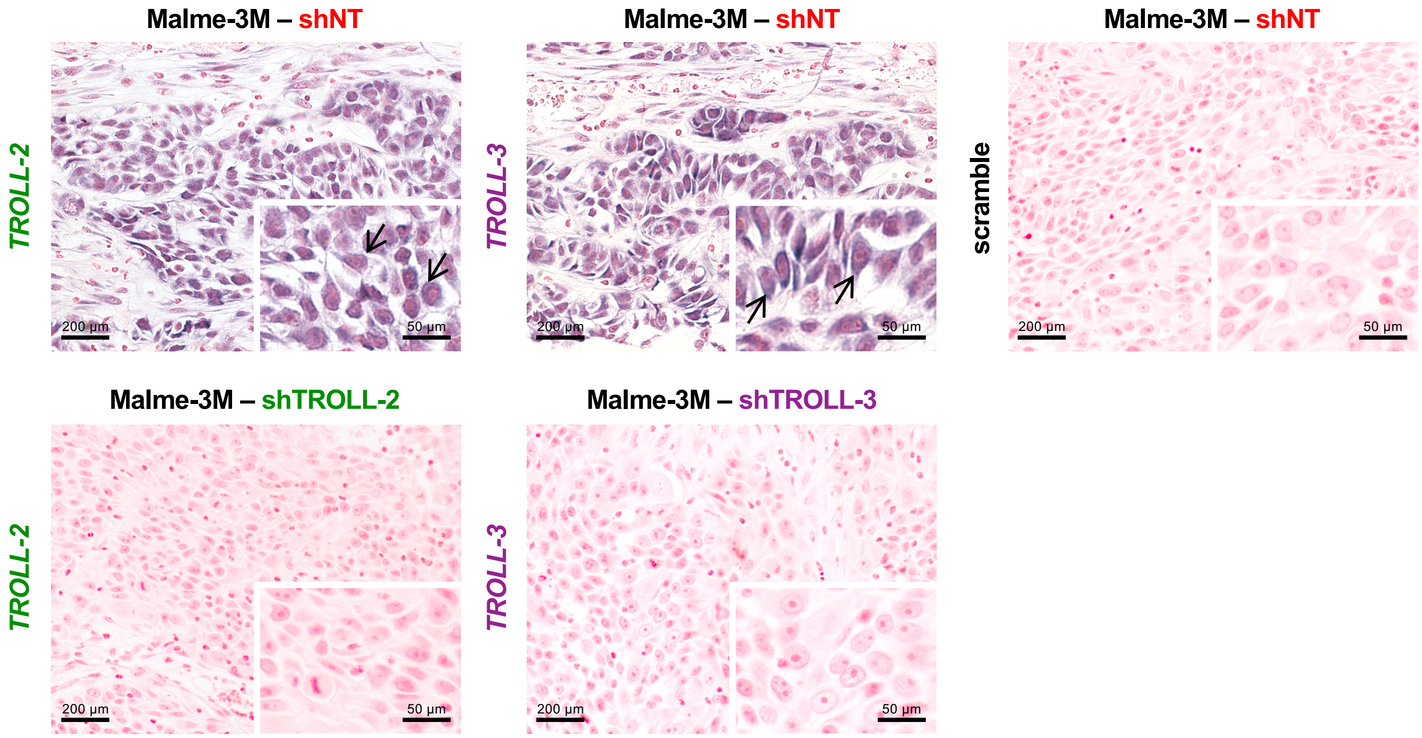
Malme-3M – shNT
Malme-3M – shNT
Malme-3M – shNT
TROLL-2
TROLL-3
scramble
200 µm
50 µm
Malme-3M – shTROLL-2
Malme-3M – shTROLL-3
TROLL-2
TROLL-3

Supplementary Fig. 5 Cytoplasmic WDR26 correlates with advanced cancers. **a-c** Quantification of the ISH scores of *TROLL-2* (**a**) and *TROLL-3* (**b**), and of the IHC score of WDR26 (**c**) in the indicated TMA. Data were analysed with two-way ANOVA. * vs. normal ovarian tissue, $P < 0.005$. § vs. serous adenocarcinoma, $P < 0.005$. **d,e** Correlation of the IHC score and cellular distribution of WDR26 with the ISH score of *TROLL-2* (**d**) and *TROLL*-3 (**e**) in the same TMA as in (**a-c**). **f-h** Quantification of the ISH scores of *TROLL-2* (**f**) and *TROLL-3* (**g**), and of the IHC score of WDR26 (**h**) in the indicated TMA. Data were analysed with two-way ANOVA. * vs. normal colon tissue, $P < 0.005$. § vs. polyp, $P < 0.005$. # vs. adenoma, $P < 0.005$. **i,j** Correlation of the IHC score and cellular distribution of WDR26 with the ISH score of *TROLL-2* (**i**) and *TROLL-3* (**j**) in the same TMA as in (**f-h**). **k-m** Quantification of the ISH scores of *TROLL-2* (**k**) and *TROLL-3* (**l**), and of the IHC score of WDR26 (**m**) in the indicated TMA. Data were analysed with two-way ANOVA. * vs. normal lung tissue, $P < 0.005$. § vs. benign lesion, $P < 0.005$. **n,o** Correlation of the IHC score and cellular distribution of WDR26 with the ISH score of *TROLL-2* (**n**) and *TROLL-3* (**o**) in the same TMA as in (**k-m**). **p-r** Quantification of the ISH scores of *TROLL-2* (**p**) and *TROLL-3* (**q**), and of the IHC score of WDR26 (**r**) in the indicated TMA. Data were analysed with two-way ANOVA. * vs. normal lung tissue, $P < 0.005$. § vs. benign lesion, $P < 0.005$. # vs. squamous cell carcinoma, $P < 0.005$. **s,t** Correlation of the IHC score and cellular distribution of WDR26 with the ISH score of *TROLL-2* (**s**) and *TROLL-3* (**t**) in the same TMA as in (**p-r**). **u-w** Quantification of the ISH scores of *TROLL-2* (**u**) and *TROLL-3* (**v**), and of the IHC score of WDR26 (**w**) in the indicated TMA. Data were analysed with two-way ANOVA. * vs. benign nevus, $P < 0.005$. **y,z** Correlation of the IHC score and cellular distribution of WDR26 with the ISH score of *TROLL-2* (**y**) and *TROLL-3* (**z**) in the same TMA as in (**u-w**). **a'-c'** Quantification of the ISH scores of *TROLL-2* (**a'**) and *TROLL-3* (**b'**) and of the IHC scores of WDR26 (**c'**) in the indicated TMA. Data were analysed with two-way ANOVA. * vs. control cases, $P < 0.005$. **d',e'** Correlation of the IHC score and cellular distribution of WDR26 with the ISH score of *TROLL-2* (**d'**) and *TROLL-3* (**e'**) in the TMA as in (**a'-c'**). **f',g'** Kaplan-Meier curves of overall survival in the indicated TMA based on the levels of *TROLL-2* (**f'**) and *TROLL-3* (**g'**). COXPH *P* values are shown. **h',i'** Kaplan-Meier curves of overall survival in the indicated TMA based on the levels of *TROLL-2* (**h'**) and *TROLL-3* (**i'**). COXPH *P* values are shown. **j',k'** qRT-PCR for *TROLL-2* (**j'**) and *TROLL-3* (**k'**) in H1299 cells infected with the indicated shRNAs. Data are mean ± SD, $n = 3$ biological replicates, * vs. shNT no doxy, $P < 0.005$, two-tailed Student's *t* test. **l',m'** qRT-PCR for *TROLL-2* (**l'**) and *TROLL-3* (**m'**) in H358 cells infected with the indicated shRNAs. Data are mean ± SD, $n = 3$ biological replicates, * vs. shNT no doxy, $P < 0.005$, two-tailed Student's *t* test. **n'** Representative H&E stained cross sections of lung adenocarcinomas derived from H1299 cells infected with the indicated shRNAs. **i** Quantification of the lung adenocarcinomas described in (**n'**). $n = 5$ mice for all groups, * vs. NT, $P < 0.005$, two-tailed Student's *t* test. **p'** Representative H&E stained cross sections of lung adenocarcinomas derived from H358 cells infected with the indicated shRNAs. **k** Quantification of the lung adenocarcinomas described in (**p'**). $n = 5$ mice for all groups, * vs. NT, $P < 0.005$, two-tailed Student's *t* test. **r'** Representative H&E stained cross sections of lung colonies derived from H1299 cells infected with the indicated shRNAs. **s'** Quantification of the lung colonies described in (**r'**). $n = 5$ mice for all groups, * vs. NT, $P < 0.005$, two-tailed Student's *t* test. **t'** Representative H&E stained cross sections of lung colonies derived from H358 cells infected with the indicated shRNAs. **u'** Quantification of the lung colonies described in (**t'**). $n = 5$ mice for all groups, * vs. NT, $P < 0.005$, two-tailed Student's *t* test. **v'** Representative images of ISH for *TROLL-2* (left panels) and *TROLL-3* (middle panels) in tumours derived from H1299 cells. **w'** Representative images of ISH for *TROLL-2* (left panels) and *TROLL-3* (middle panels) in tumours derived from H358 cells. **x',y'** qRT-PCR for *TROLL-2* (**x'**) and *TROLL-3* (**y'**) in A375 cells infected with the indicated shRNAs. Data are mean ± SD, $n = 3$ biological replicates, * vs. shNT no doxy, $P < 0.005$, two-tailed Student's *t* test. **z',a''** qRT-PCR for *TROLL-2* (**z'**) and *TROLL-3* (**z''**) in Malme-3M cells infected with the indicated shRNAs. Data are mean ± SD, $n = 3$ biological replicates, * vs. shNT no doxy, $P < 0.005$, two-tailed Student's *t* test. **f''** Representative images of ISH for *TROLL-2* (left panels) and *TROLL-3* (middle panels) in tumours derived from A375 cells. **g''** Representative images of ISH for *TROLL-2* (left panels) and *TROLL-3* (middle panels) in tumours derived from Malme-3M cells. Micrographs are representative of $n = 10$ tumours. All boxplots represent the individual data points, median and whiskers (min to max method). Source data are provided as Source data file.

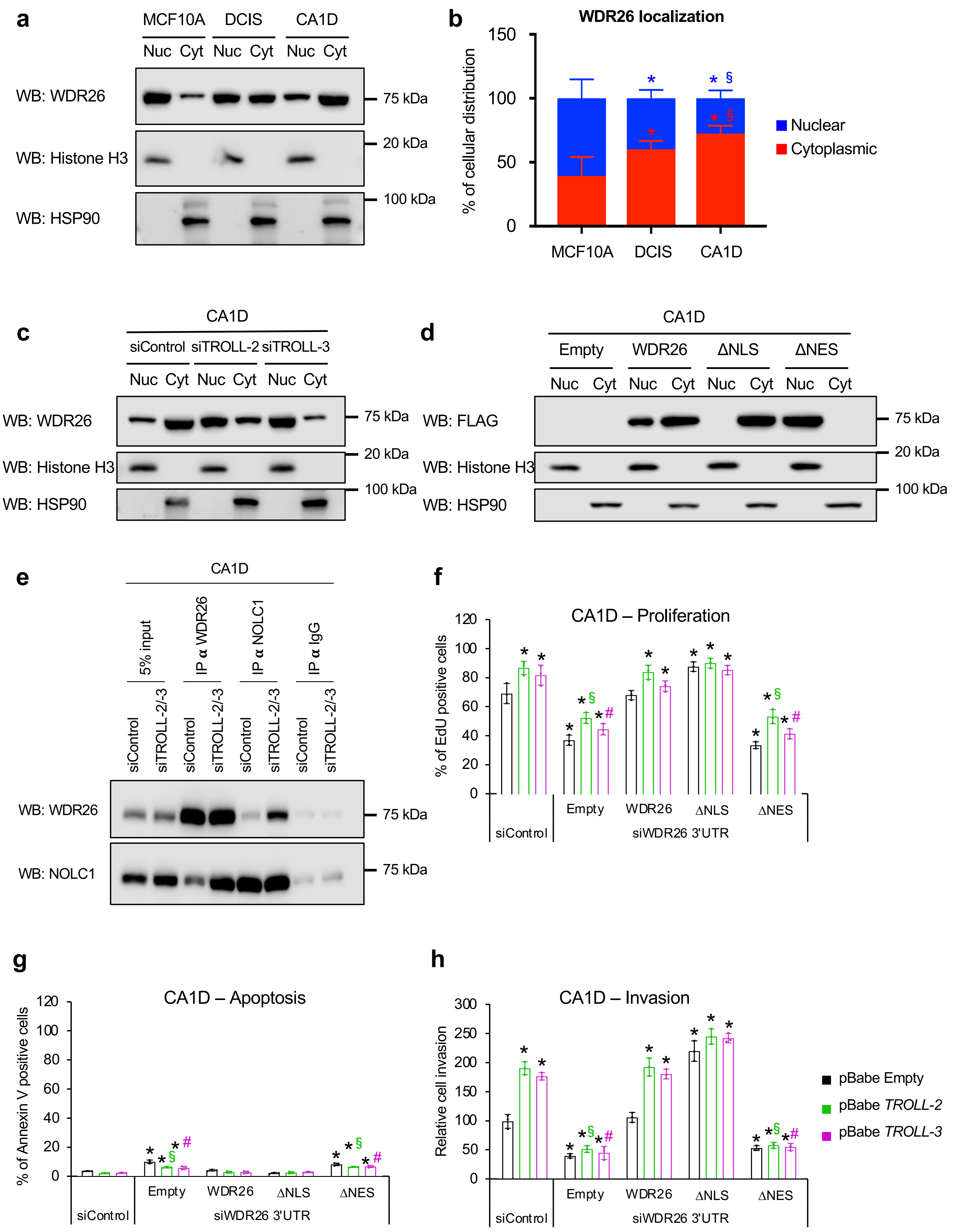

a
MCF10A DCIS CA1D
Nuc Cyt Nuc Cyt Nuc Cyt
WB: WDR26
WB: Histone H3
WB: HSP90
75 kDa
20 kDa
100 kDa
b
WDR26 localization
% of cellular distribution
Nuclear
Cytoplasmic
MCF10A DCIS CA1D
c
CA1D
siControl siTROLL-2 siTROLL-3
d
Empty WDR26 ΔNLS ΔNES
WB: FLAG
e
5% input
IP α WDR26
IP α NOLC1
IP α IgG
siControl
siTROLL-2/-3
WB: NOLC1
f
CA1D – Proliferation
% of EdU positive cells
siWDR26 3'UTR
g
CA1D – Apoptosis
% of Annexin V positive cells
h
CA1D – Invasion
Relative cell invasion
pBabe Empty
pBabe TROLL-2
pBabe TROLL-3

i

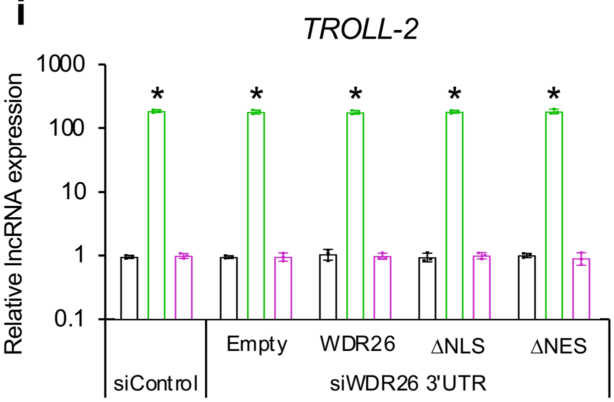

TROLL-2
Relative lncRNA expression
1000
100
10
1
0.1
*
Empty
WDR26
ΔNLS
ΔNES
siControl
siWDR26 3'UTR

j

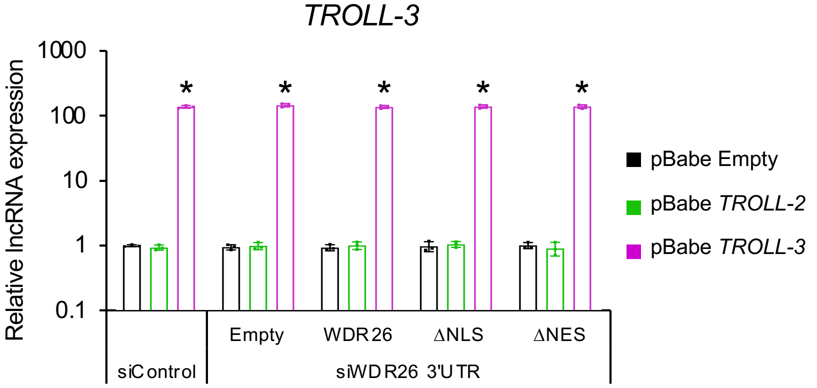

TROLL-3
Relative lncRNA expression
1000
100
10
1
0.1
*
Empty
WDR26
ΔNLS
ΔNES
siControl
siWDR26 3'UTR
pBabe Empty
pBabe TROLL-2
pBabe TROLL-3

k

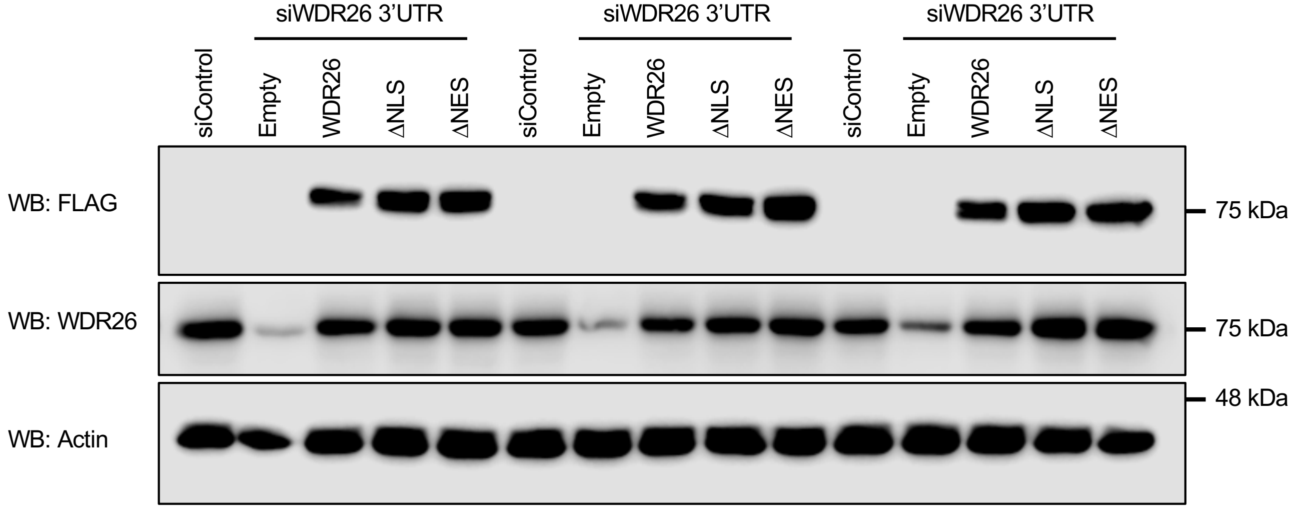

siWDR26 3'UTR
siWDR26 3'UTR
siWDR26 3'UTR
siControl
Empty
WDR26
ΔNLS
ΔNES
siControl
Empty
WDR26
ΔNLS
ΔNES
siControl
Empty
WDR26
ΔNLS
ΔNES
WB: FLAG
WB: WDR26
WB: Actin
75 kDa
75 kDa
48 kDa

l

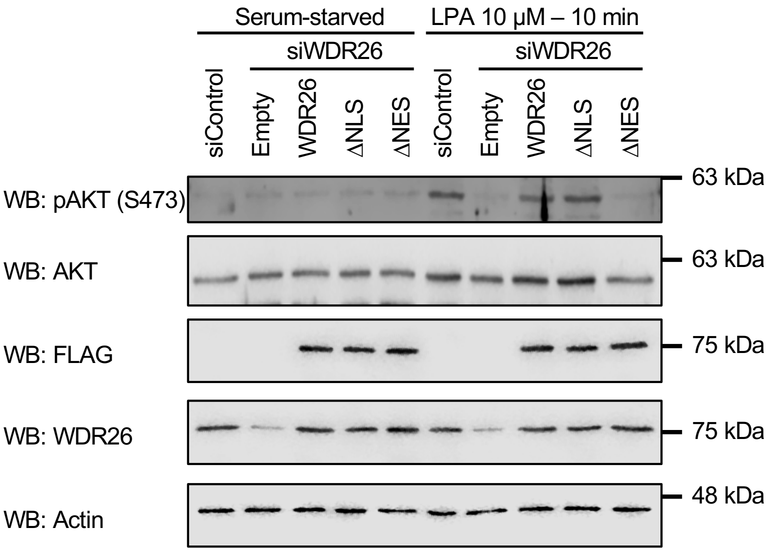

Serum-starved
LPA 10 μM – 10 min
siWDR26
siWDR26
siControl
Empty
WDR26
ΔNLS
ΔNES
siControl
Empty
WDR26
ΔNLS
ΔNES
WB: pAKT (S473)
WB: AKT
WB: FLAG
WB: WDR26
WB: Actin
63 kDa
63 kDa
75 kDa
75 kDa
48 kDa

m

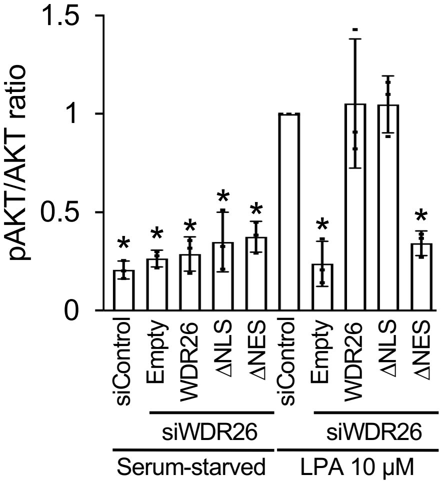

pAKT/AKT ratio
1.5
1
0.5
0
*
siControl
Empty
WDR26
ΔNLS
ΔNES
siControl
Empty
WDR26
ΔNLS
ΔNES
siWDR26
siWDR26
Serum-starved
LPA 10 μM

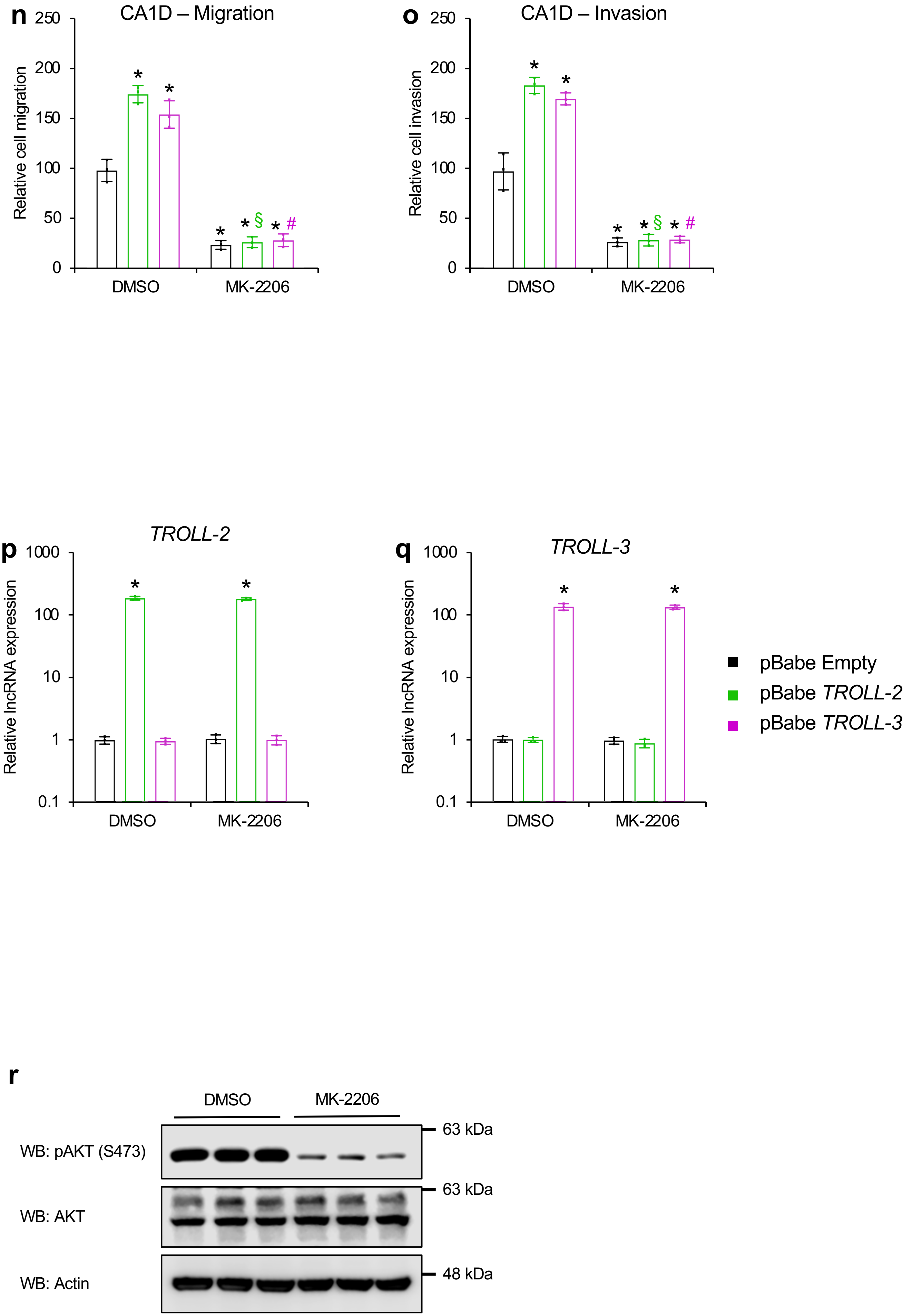
n
CA1D – Migration
Relative cell migration
250
200
150
100
50
0
DMSO
MK-2206
o
CA1D – Invasion
Relative cell invasion
DMSO
MK-2206
p
TROLL-2
Relative lncRNA expression
1000
100
10
1
0.1
DMSO
MK-2206
q
TROLL-3
Relative lncRNA expression
DMSO
MK-2206
pBabe Empty
pBabe TROLL-2
pBabe TROLL-3
r
DMSO
MK-2206
WB: pAKT (S473)
WB: AKT
WB: Actin
63 kDa
63 kDa
48 kDa

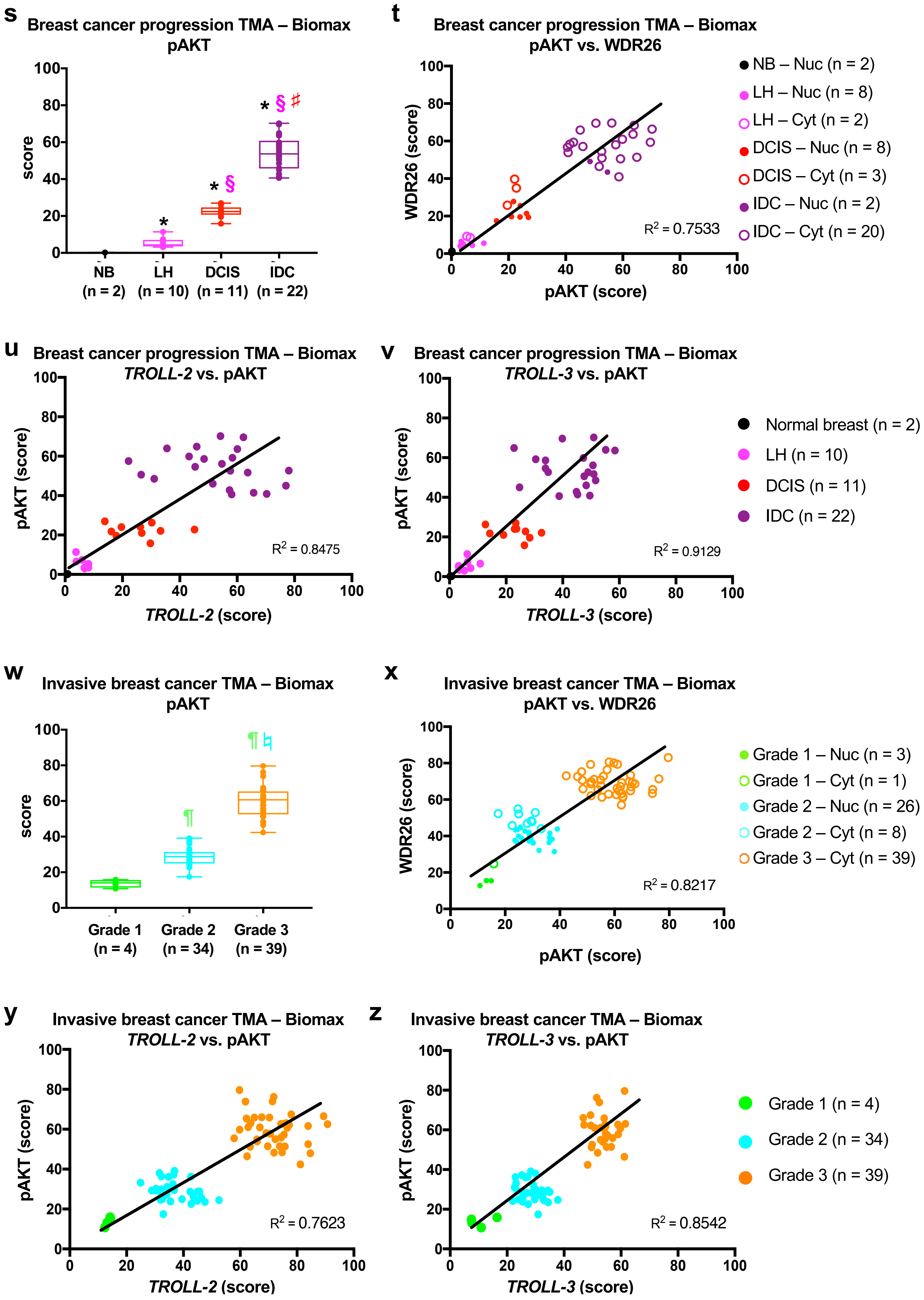
s
Breast cancer progression TMA – Biomax
pAKT
score
NB (n = 2)
LH (n = 10)
DCIS (n = 11)
IDC (n = 22)
t
Breast cancer progression TMA – Biomax
pAKT vs. WDR26
WDR26 (score)
pAKT (score)
R2 = 0.7533
NB – Nuc (n = 2)
LH – Nuc (n = 8)
LH – Cyt (n = 2)
DCIS – Nuc (n = 8)
DCIS – Cyt (n = 3)
IDC – Nuc (n = 2)
IDC – Cyt (n = 20)
u
Breast cancer progression TMA – Biomax
TROLL-2 vs. pAKT
pAKT (score)
TROLL-2 (score)
R2 = 0.8475
v
Breast cancer progression TMA – Biomax
TROLL-3 vs. pAKT
pAKT (score)
TROLL-3 (score)
R2 = 0.9129
Normal breast (n = 2)
LH (n = 10)
DCIS (n = 11)
IDC (n = 22)
w
Invasive breast cancer TMA – Biomax
pAKT
score
Grade 1 (n = 4)
Grade 2 (n = 34)
Grade 3 (n = 39)
x
Invasive breast cancer TMA – Biomax
pAKT vs. WDR26
WDR26 (score)
pAKT (score)
R2 = 0.8217
Grade 1 – Nuc (n = 3)
Grade 1 – Cyt (n = 1)
Grade 2 – Nuc (n = 26)
Grade 2 – Cyt (n = 8)
Grade 3 – Cyt (n = 39)
y
Invasive breast cancer TMA – Biomax
TROLL-2 vs. pAKT
pAKT (score)
TROLL-2 (score)
R2 = 0.7623
z
Invasive breast cancer TMA – Biomax
TROLL-3 vs. pAKT
pAKT (score)
TROLL-3 (score)
R2 = 0.8542
Grade 1 (n = 4)
Grade 2 (n = 34)
Grade 3 (n = 39)

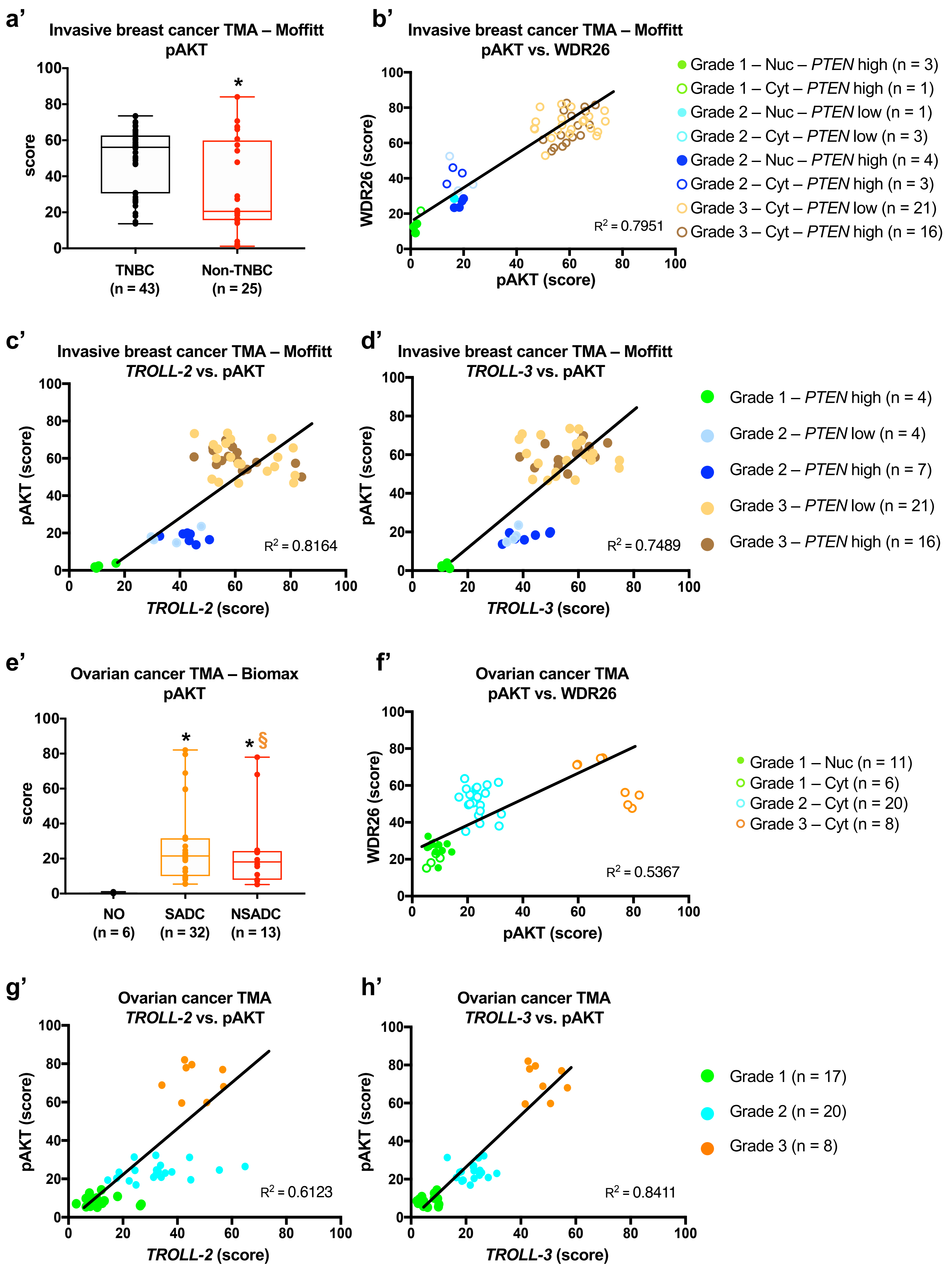

a'
Invasive breast cancer TMA – Moffitt
pAKT
score
TNBC (n = 43)
Non-TNBC (n = 25)
b'
Invasive breast cancer TMA – Moffitt
pAKT vs. WDR26
WDR26 (score)
pAKT (score)
R2 = 0.7951
Grade 1 – Nuc – PTEN high (n = 3)
Grade 1 – Cyt – PTEN high (n = 1)
Grade 2 – Nuc – PTEN low (n = 1)
Grade 2 – Cyt – PTEN low (n = 3)
Grade 2 – Nuc – PTEN high (n = 4)
Grade 2 – Cyt – PTEN high (n = 3)
Grade 3 – Cyt – PTEN low (n = 21)
Grade 3 – Cyt – PTEN high (n = 16)
c'
Invasive breast cancer TMA – Moffitt
TROLL-2 vs. pAKT
pAKT (score)
TROLL-2 (score)
R2 = 0.8164
d'
Invasive breast cancer TMA – Moffitt
TROLL-3 vs. pAKT
pAKT (score)
TROLL-3 (score)
R2 = 0.7489
Grade 1 – PTEN high (n = 4)
Grade 2 – PTEN low (n = 4)
Grade 2 – PTEN high (n = 7)
Grade 3 – PTEN low (n = 21)
Grade 3 – PTEN high (n = 16)
e'
Ovarian cancer TMA – Biomax
pAKT
score
NO (n = 6)
SADC (n = 32)
NSADC (n = 13)
f'
Ovarian cancer TMA
pAKT vs. WDR26
WDR26 (score)
pAKT (score)
R2 = 0.5367
Grade 1 – Nuc (n = 11)
Grade 1 – Cyt (n = 6)
Grade 2 – Cyt (n = 20)
Grade 3 – Cyt (n = 8)
g'
Ovarian cancer TMA
TROLL-2 vs. pAKT
pAKT (score)
TROLL-2 (score)
R2 = 0.6123
h'
Ovarian cancer TMA
TROLL-3 vs. pAKT
pAKT (score)
TROLL-3 (score)
R2 = 0.8411
Grade 1 (n = 17)
Grade 2 (n = 20)
Grade 3 (n = 8)

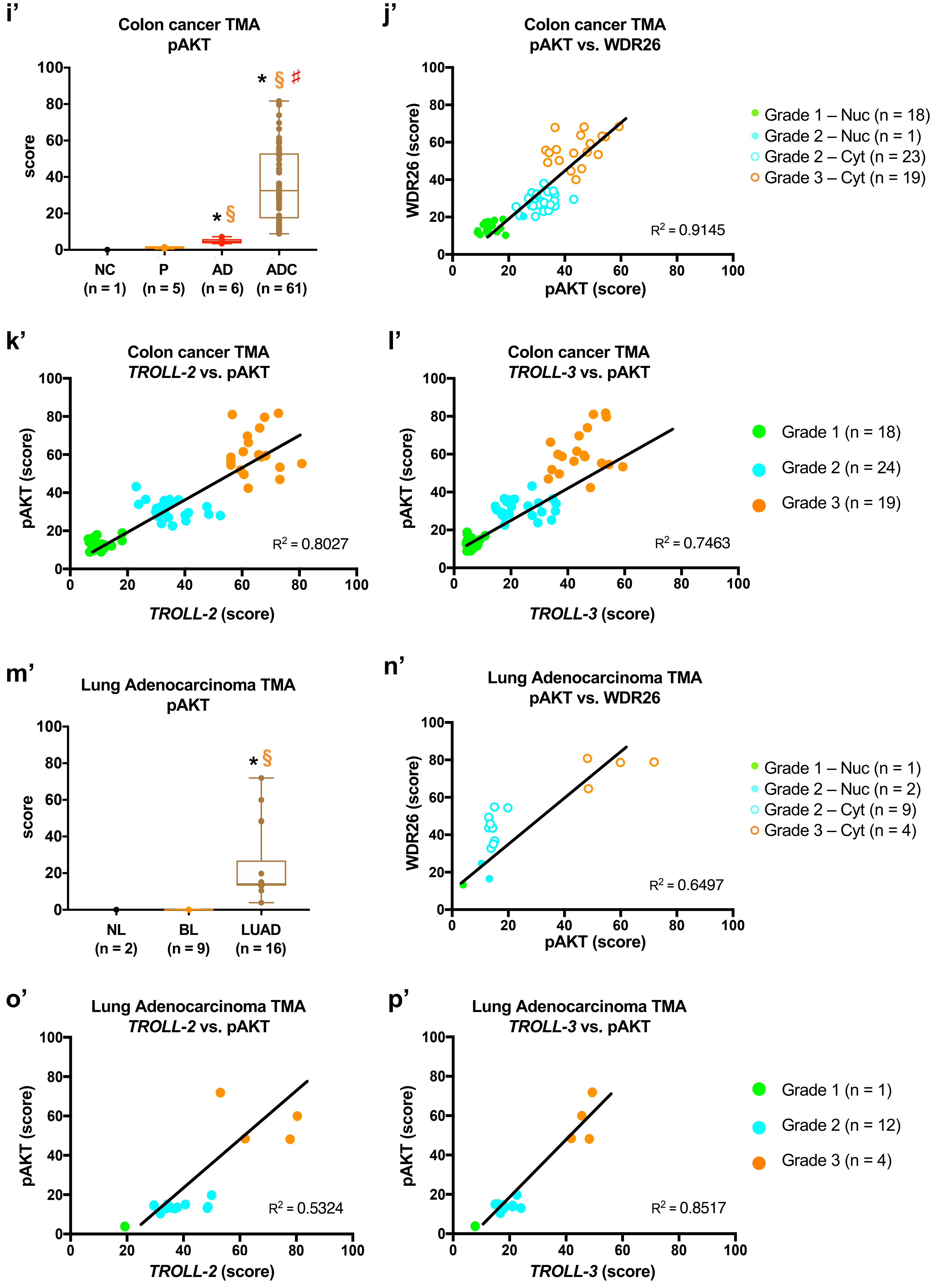
i'
Colon cancer TMA
pAKT
score
NC (n = 1)
P (n = 5)
AD (n = 6)
ADC (n = 61)
* §
* § ♯
j'
Colon cancer TMA
pAKT vs. WDR26
WDR26 (score)
pAKT (score)
R² = 0.9145
Grade 1 – Nuc (n = 18)
Grade 2 – Nuc (n = 1)
Grade 2 – Cyt (n = 23)
Grade 3 – Cyt (n = 19)
k'
Colon cancer TMA
TROLL-2 vs. pAKT
pAKT (score)
TROLL-2 (score)
R² = 0.8027
l'
Colon cancer TMA
TROLL-3 vs. pAKT
pAKT (score)
TROLL-3 (score)
R² = 0.7463
Grade 1 (n = 18)
Grade 2 (n = 24)
Grade 3 (n = 19)
m'
Lung Adenocarcinoma TMA
pAKT
score
NL (n = 2)
BL (n = 9)
LUAD (n = 16)
* §
n'
Lung Adenocarcinoma TMA
pAKT vs. WDR26
WDR26 (score)
pAKT (score)
R² = 0.6497
Grade 1 – Nuc (n = 1)
Grade 2 – Nuc (n = 2)
Grade 2 – Cyt (n = 9)
Grade 3 – Cyt (n = 4)
o'
Lung Adenocarcinoma TMA
TROLL-2 vs. pAKT
pAKT (score)
TROLL-2 (score)
R² = 0.5324
p'
Lung Adenocarcinoma TMA
TROLL-3 vs. pAKT
pAKT (score)
TROLL-3 (score)
R² = 0.8517
Grade 1 (n = 1)
Grade 2 (n = 12)
Grade 3 (n = 4)

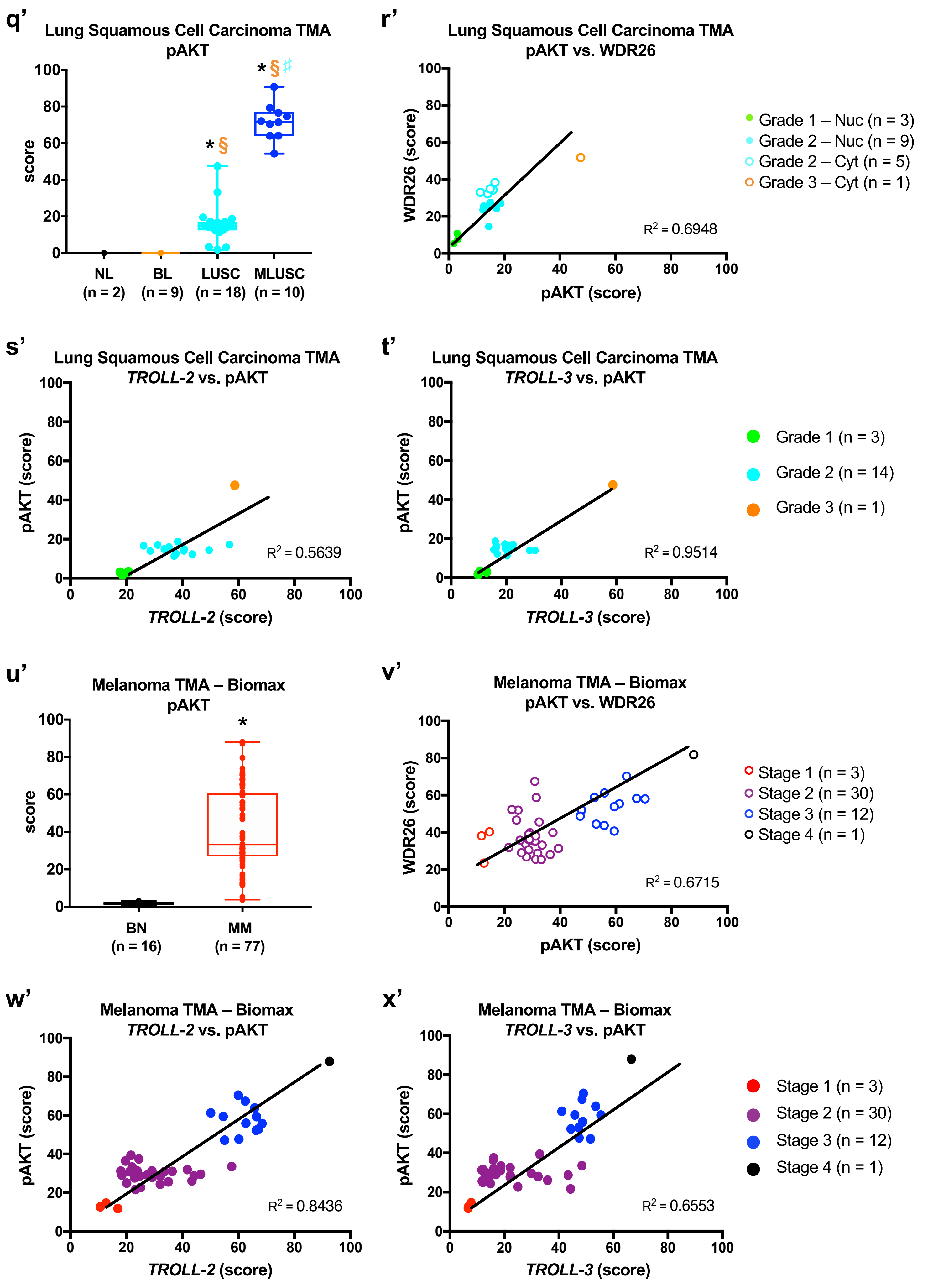
q'
Lung Squamous Cell Carcinoma TMA
pAKT
score
NL (n = 2)
BL (n = 9)
LUSC (n = 18)
MLUSC (n = 10)
r'
Lung Squamous Cell Carcinoma TMA
pAKT vs. WDR26
WDR26 (score)
pAKT (score)
R2 = 0.6948
Grade 1 – Nuc (n = 3)
Grade 2 – Nuc (n = 9)
Grade 2 – Cyt (n = 5)
Grade 3 – Cyt (n = 1)
s'
Lung Squamous Cell Carcinoma TMA
TROLL-2 vs. pAKT
pAKT (score)
TROLL-2 (score)
R2 = 0.5639
t'
Lung Squamous Cell Carcinoma TMA
TROLL-3 vs. pAKT
pAKT (score)
TROLL-3 (score)
R2 = 0.9514
Grade 1 (n = 3)
Grade 2 (n = 14)
Grade 3 (n = 1)
u'
Melanoma TMA – Biomax
pAKT
score
BN (n = 16)
MM (n = 77)
v'
Melanoma TMA – Biomax
pAKT vs. WDR26
WDR26 (score)
pAKT (score)
R2 = 0.6715
Stage 1 (n = 3)
Stage 2 (n = 30)
Stage 3 (n = 12)
Stage 4 (n = 1)
w'
Melanoma TMA – Biomax
TROLL-2 vs. pAKT
pAKT (score)
TROLL-2 (score)
R2 = 0.8436
x'
Melanoma TMA – Biomax
TROLL-3 vs. pAKT
pAKT (score)
TROLL-3 (score)
R2 = 0.6553
Stage 1 (n = 3)
Stage 2 (n = 30)
Stage 3 (n = 12)
Stage 4 (n = 1)

y’

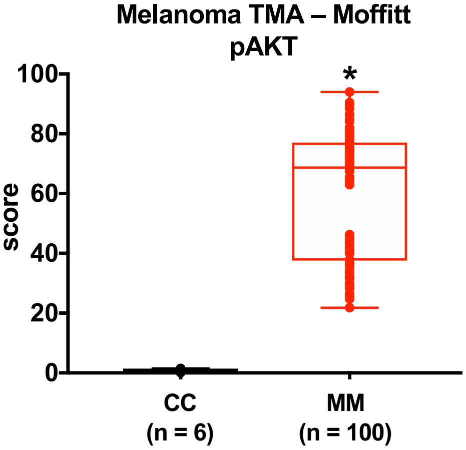

Melanoma TMA – Moffitt
pAKT
score
100
80
60
40
20
0
*
CC
(n = 6)
MM
(n = 100)

z’

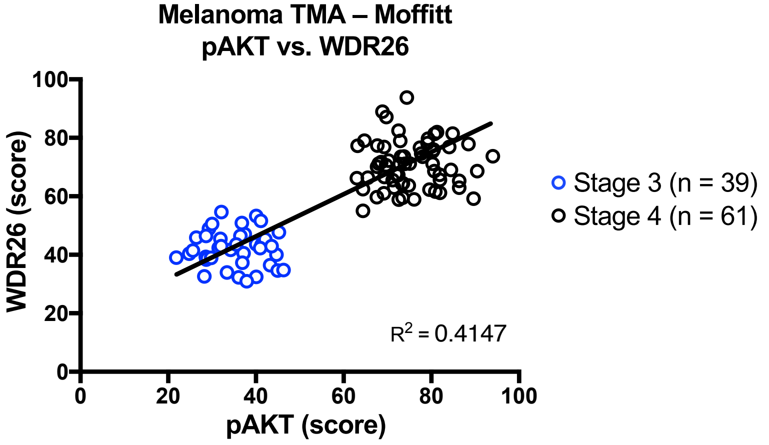

Melanoma TMA – Moffitt
pAKT vs. WDR26
WDR26 (score)
pAKT (score)
0
20
40
60
80
100
R^2 = 0.4147
Stage 3 (n = 39)
Stage 4 (n = 61)

a’’

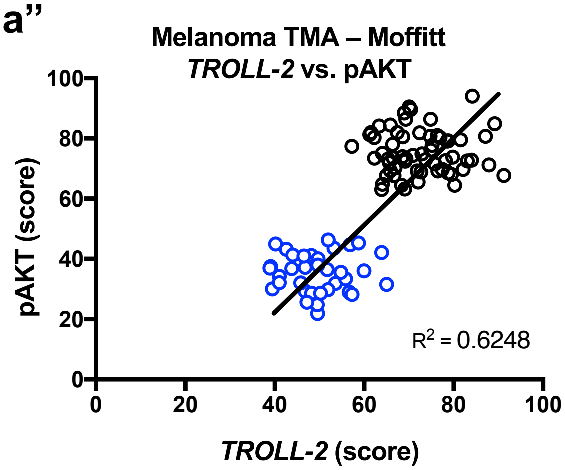

Melanoma TMA – Moffitt
TROLL-2 vs. pAKT
pAKT (score)
TROLL-2 (score)
0
20
40
60
80
100
R^2 = 0.6248

b’’

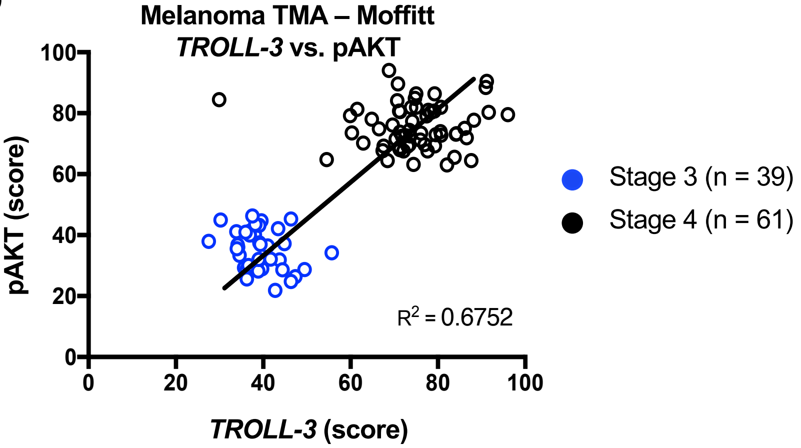

Melanoma TMA – Moffitt
TROLL-3 vs. pAKT
pAKT (score)
TROLL-3 (score)
0
20
40
60
80
100
R^2 = 0.6752
Stage 3 (n = 39)
Stage 4 (n = 61)

c’’

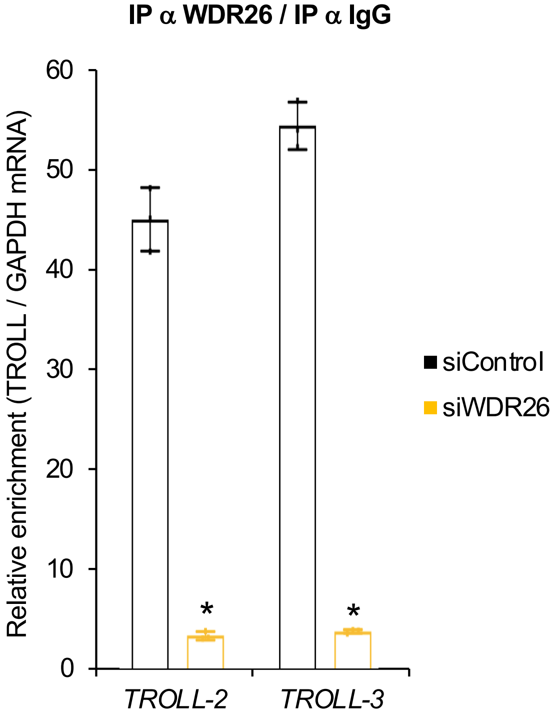

IP α WDR26 / IP α IgG
Relative enrichment (TROLL / GAPDH mRNA)
0
10
20
30
40
50
60
*
*
TROLL-2
TROLL-3
siControl
siWDR26

d’’

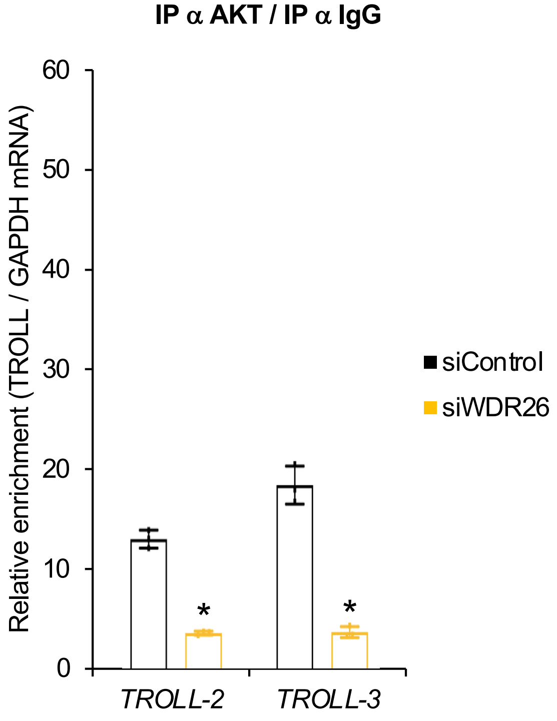

IP α AKT / IP α IgG
Relative enrichment (TROLL / GAPDH mRNA)
0
10
20
30
40
50
60
*
*
TROLL-2
TROLL-3
siControl
siWDR26

e''

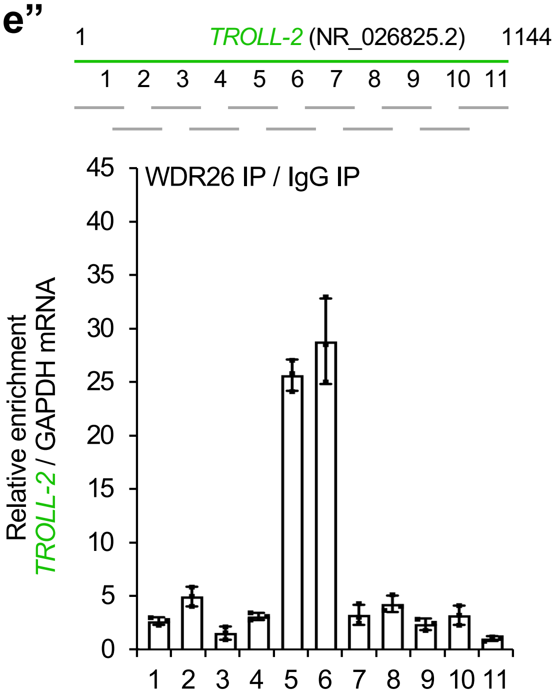
1
TROLL-2 (NR_026825.2)
1144
1 2 3 4 5 6 7 8 9 10 11
WDR26 IP / IgG IP
Relative enrichment
TROLL-2 / GAPDH mRNA
0 5 10 15 20 25 30 35 40 45
1 2 3 4 5 6 7 8 9 10 11

f''

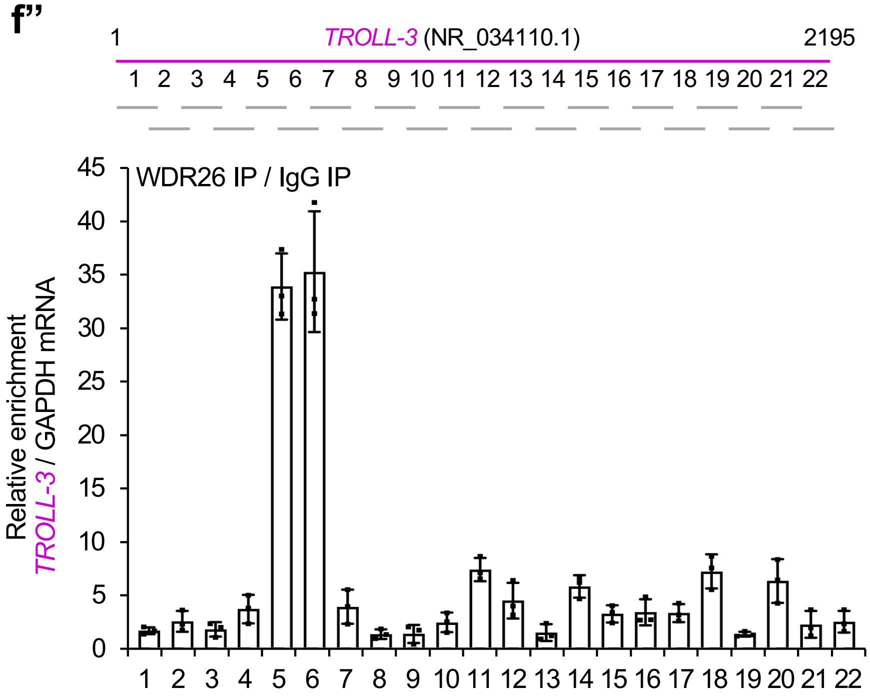
1
TROLL-3 (NR_034110.1)
2195
1 2 3 4 5 6 7 8 9 10 11 12 13 14 15 16 17 18 19 20 21 22
WDR26 IP / IgG IP
Relative enrichment
TROLL-3 / GAPDH mRNA
0 5 10 15 20 25 30 35 40 45
1 2 3 4 5 6 7 8 9 10 11 12 13 14 15 16 17 18 19 20 21 22

g''

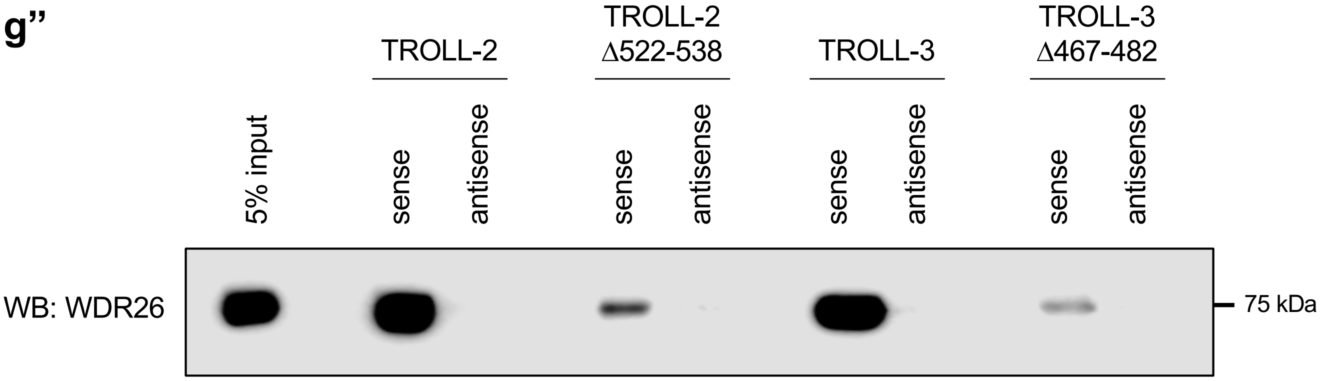
TROLL-2
TROLL-2 Δ522-538
TROLL-3
TROLL-3 Δ467-482
5% input
sense
antisense
sense
antisense
sense
antisense
sense
antisense
WB: WDR26
75 kDa

h''

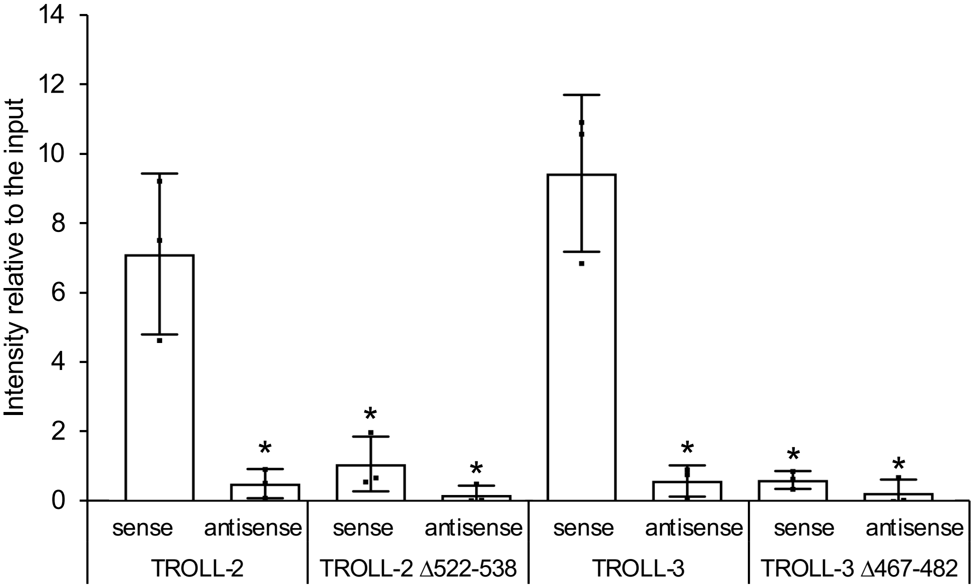
Intensity relative to the input
0 2 4 6 8 10 12 14
*
sense
antisense
TROLL-2
sense
antisense
TROLL-2 Δ522-538
sense
antisense
TROLL-3
sense
antisense
TROLL-3 Δ467-482

i''

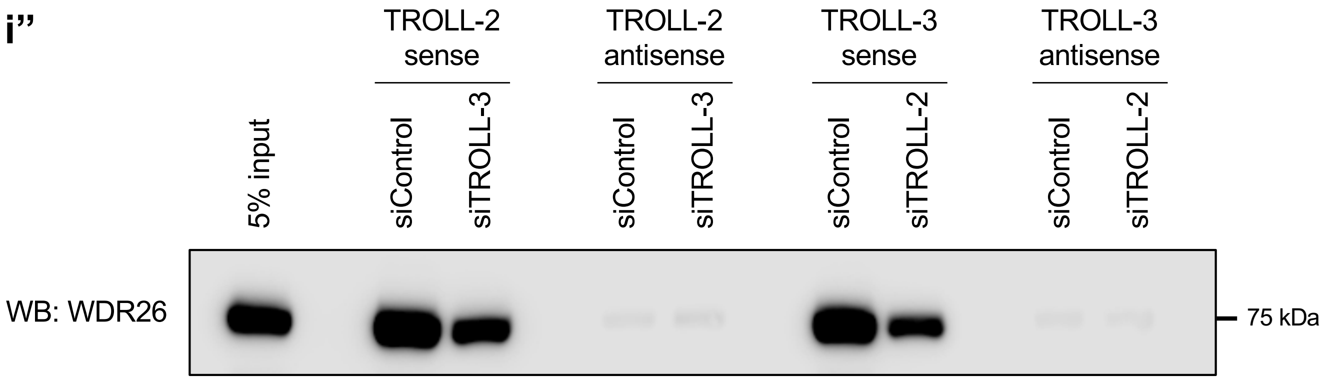

TROLL-2 sense
TROLL-2 antisense
TROLL-3 sense
TROLL-3 antisense
5% input
siControl
siTROLL-3
siControl
siTROLL-3
siControl
siTROLL-2
siControl
siTROLL-2
WB: WDR26
75 kDa

j''

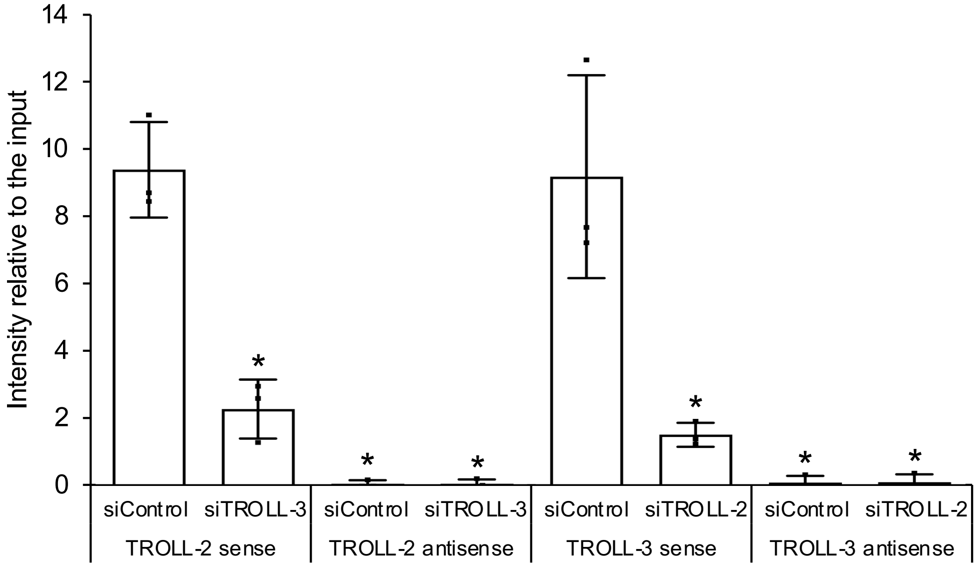

Intensity relative to the input
0
2
4
6
8
10
12
14
*
siControl
siTROLL-3
siControl
siTROLL-3
siControl
siTROLL-2
siControl
siTROLL-2
TROLL-2 sense
TROLL-2 antisense
TROLL-3 sense
TROLL-3 antisense

k''

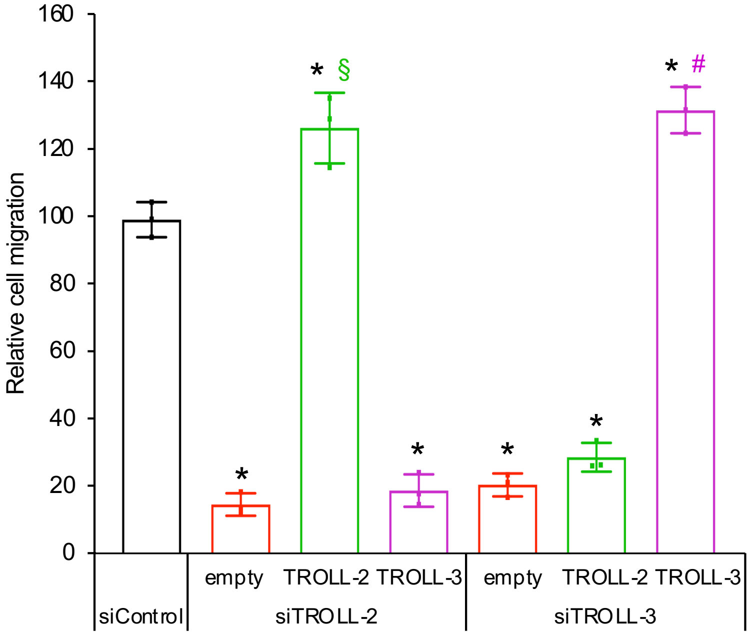

Relative cell migration
0
20
40
60
80
100
120
140
160
* §
* #
*
siControl
empty
TROLL-2
TROLL-3
empty
TROLL-2
TROLL-3
siTROLL-2
siTROLL-3

l''

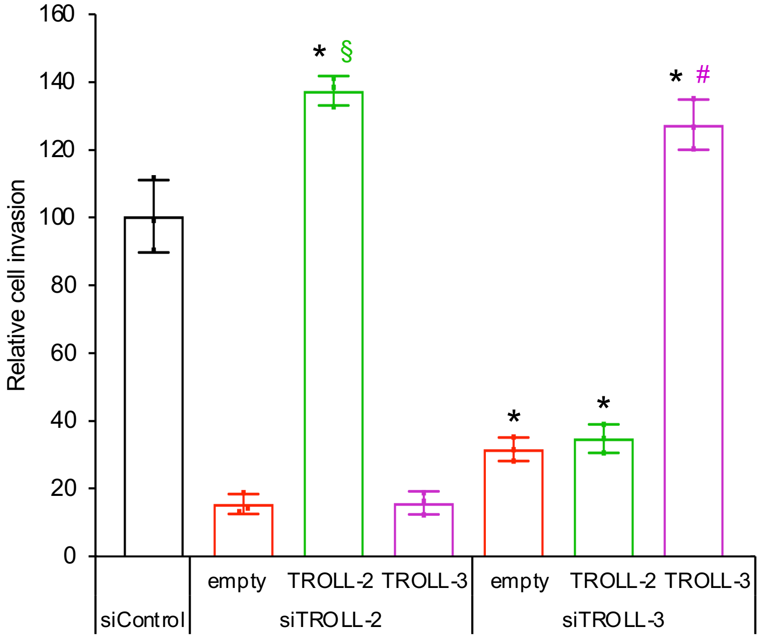

Relative cell invasion
0
20
40
60
80
100
120
140
160
* §
* #
*
siControl
empty
TROLL-2
TROLL-3
empty
TROLL-2
TROLL-3
siTROLL-2
siTROLL-3

Supplementary Fig. 6 AKT phosphorylation mediates *TROLL-2* and *TROLL-3*'s functions. **a**,**b** Representative western blot (**a**) and quantification of the percentage (**b**) of WDR26 localization in the nuclei (Nuc) and cytosols (Cyt) of the indicated cell lines. Data are mean ± SD and analysed with two-way ANOVA. *n* = 3 biological replicates, * vs. MCF10A, *P* < 0.05. § vs. DCIS, *P* < 0.005. **c** Representative western blot of WDR26 localization in the nuclear (Nuc) and cytoplasmic (Cyt) fractions of CA1D cells transfected with the indicated siRNAs. **d** Representative western blot of WDR26 localization in fractionated CA1D cells transfected with the indicated constructs. **e** Representative western blot analysis of the coimmuno-precipitation of endogenous NOLC1 and WDR26 in CA1D cells transfected with the indicated constructs and siRNAs. **f-h** Quantification for EdU incorporation (**f**), annexin V positivity (**g**), and cell invasion (**h**) of CA1D cells overexpressing either *TROLL-2*, *TROLL-3*, or the empty vector as a negative control, in combination with the indicated WDR26 constructs and transfected with the indicated siRNAs. Data are mean ± SD and analysed with two-tailed Student's *t* test, *n* = 3 biological replicates, * vs. pBabe Empty siControl, *P* < 0.005. § vs. pBabe *TROLL-2* siControl, *P* < 0.005. # vs. pBabe *TROLL-3* siControl, *P* < 0.005. **i**,**j** qRT-PCR for *TROLL-2* (**i**) and *TROLL-3* (**j**) of the CA1D cells treated as in (**f-h**). Data are mean ± SD and analysed with two-tailed Student's *t* test, *n* = 3, * vs. pBabe Empty siControl, *P* < 0.005. **k** Representative western blot of WDR26 and FLAG in the CA1D cells treated as in (**f-h**). **l,m** Representative western blot (**l**) and quantification of the ratio between pAKT (S473) and total AKT in western blots of LPA-treated CA1D cells transfected with the indicated siRNAs and WDR26 constructs. Data are mean ± SD and analysed with two-tailed Student's *t* test, *n* = 3 biological replicates, * vs. siControl – LPA treatment, *P* < 0.005. **n,o** Quantification of cell migration (**n**) and invasion (**o**) of CA1D cells overexpressing either *TROLL-2*, *TROLL-3*, or the empty vector, and treated with either MK-2206 or DMSO. Data are mean ± SD and analysed with two-tailed Student's *t* test, *n* = 3 biological replicates, * vs. pBabe Empty siControl, *P* < 0.005. **p,q** qRT-PCR for *TROLL-2* (**p**) and *TROLL-3* (**q**) in CA1D cells treated as in (**n,o**). Data are mean ± SD and analysed with two-tailed Student's *t* test, *n* = 3, * vs. pBabe Empty siControl, *P* < 0.005. **r** Representative western blot of the indicated proteins in the CA1D cells treated as in (**n,o**). **s** Quantification of the IHC scores of pAKT in the indicated TMA. Data were analysed with two-way ANOVA. * vs. normal breast tissue, *P* < 0.005. § vs. lobular hyperplasia, *P* < 0.005. # vs. ductal carcinoma in situ, *P* < 0.005. **t-v** Correlation of the IHC score of pAKT with the IHC score and cellular distribution of WDR26 (**t**), and with the ISH score of *TROLL-2* (**u**) and *TROLL-3* (**v**) in the indicated TMA. **w** Quantification of the IHC scores of pAKT in the indicated TMA. Data were analysed with two-way ANOVA. ¶ vs. grade 1, *P* < 0.005. ♮ vs. grade 2, *P* < 0.005. **x-z** Correlation of the IHC score of pAKT with the IHC score and cellular distribution of WDR26 (**x**), and with the ISH score of *TROLL-2* (**y**) and *TROLL-3* (**z**) in the indicated TMA. **a'** Quantification of the IHC scores of pAKT in the indicated TMA. * vs. TNBC, *P* < 0.005, Welchs *t* test. **b'-d'** Correlation of the IHC score of pAKT with the IHC score and cellular distribution of WDR26 (**b'**), and with the ISH score of *TROLL-2* (**c'**) and *TROLL-3* (**d'**) in the indicated TMA, based on tumour grade and *PTEN* levels. **e'** Quantification of the IHC score of pAKT in the indicated TMA. Data were analysed with two-way ANOVA. * vs. normal ovarian tissue, *P* < 0.005. § vs. serous adenocarcinoma, *P* < 0.005. **f'-h'** Correlation of the IHC score of pAKT with the IHC score and cellular distribution of WDR26 (**f'**), and with the ISH score of *TROLL-2* (**g'**) and *TROLL-3* (**h'**) in the indicated TMA. **i'** Quantification of the IHC score of pAKT in the indicated TMA. Data were analysed with two-way ANOVA. * vs. normal colon tissue, *P* < 0.005. § vs. polyp, *P* < 0.005. # vs. adenoma, *P* < 0.005. **j'-l'** Correlation of the IHC score of pAKT with the IHC score and cellular distribution of WDR26 (**j'**), and with the ISH score of *TROLL-2* (**k'**) and *TROLL-3* (**l'**) in the indicated TMA. **m'** Quantification of the IHC score of pAKT in the indicated TMA. Data were analysed with two-way ANOVA. * vs. normal lung tissue, *P* < 0.005. § vs. benign lesion, *P* < 0.005. **n'-p'** Correlation of the IHC score of pAKT with the IHC score and cellular distribution of WDR26 (**n'**), and with the ISH score of *TROLL-2* (**o'**) and *TROLL-3* (**p'**) in the indicated TMA. **q'** Quantification of the IHC score of pAKT in the indicated TMA. Data were analysed with two-way ANOVA. * vs. normal lung tissue, *P* < 0.005. § vs. benign lesion, *P* < 0.005. # vs. squamous cell carcinoma, *P* < 0.005. **r'-t'** Correlation of the IHC score of pAKT with the IHC score and cellular distribution of WDR26 (**r'**), and with the ISH score of *TROLL-2* (**s'**) and *TROLL-3* (**t'**) in the indicated TMA. **u'** Quantification of the IHC score of pAKT in the indicated TMA. Data were analysed with two-way ANOVA. * vs. benign nevus, *P* < 0.005. **v'-x'** Correlation of the IHC score of pAKT with the IHC score and cellular distribution of WDR26 (**v'**), and with the ISH score of *TROLL-2* (**w'**) and *TROLL-3* (**x'**) in the indicated melanoma TMA. **y'** Quantification of the IHC score of pAKT in the indicated TMA. Data were analysed with two-way ANOVA. * vs. control cases, *P* < 0.005. **z'-b"** Correlation of the IHC score of pAKT with the IHC score and cellular distribution of WDR26 (**z'**), and with the ISH score of *TROLL-2* (**a"**) and *TROLL-3* (**b"**) in the indicated TMA. **c",d"** qRT-PCR of the indicated lncRNAs in the RNase-free CLIP-ed RNA interacting with endogenous WDR6 (**c"**) and AKT (**d"**) immunoprecipitated from CA1D cells transfected with the indicated siRNAs. Data are mean ± SD, *n* = 3 biological replicates, * vs. siControl, *P* < 0.005, two-tailed Student's *t* test. **e",f"** qRT-PCR of the indicated regions of *TROLL-2* (**e"**) and *TROLL-3* (**f"**) in the RNase-treated CLIP-ed RNA interacting with endogenous WDR6 immunoprecipitated from CA1D cells. Data are mean ± SD, *n* = 3 biological replicates. **g",h"** Representative western blot (**g"**) and quantification (**h"**) of endogenous WDR26 pulled down from CA1D cells by the indicated lncRNAs. Data are mean ± SD, *n* = 3 biological replicates, * vs. the sense strand of the respective full-length lncRNA, *P* < 0.005, two-tailed Student's *t* test. **i",j"** Representative western blot (**i"**) and quantification (**j"**) of endogenous WDR26 pulled down from CA1D cells by the indicated lncRNAs. Data are mean ± SD, *n* = 3 biological replicates, * vs. the sense strand of the respective full-length lncRNA, *P* < 0.005, two-tailed Student's *t* test. **k",l"** Quantification of cell migration (**k"**) and invasion (**l"**) of CA1D cells overexpressing either *TROLL-2*, *TROLL-3*, or the empty vector, and transfected with the indicated siRNAs. Data are mean ± SD and analysed with two-tailed Student's *t* test, *n* = 3 biological replicates, * vs. pBabe Empty siControl, *P* < 0.005. § vs. pBabe *TROLL-2* siControl, *P* < 0.005. # vs. pBabe *TROLL-3* siControl, *P* < 0.005. Graphs represent the individual data points, mean ± SD of 3 independent experiments. Boxplots represent the individual data points, median and whiskers (min to max method). All blots are representative of *n* = 3 biological replicates. Source data are provided as Source data file.

SUPPLEMENTARY TABLES

Supplementary Table 1 Gene expression qRT-PCR primers (Applied Biosystem).

Species	Gene Name	TaqMan Assay ID
Mouse	TROLL-1 (RP23-168E14.4)	Mm01219093_m1
Mouse	TROLL-2 (1700006J14Rik)	Mm01164814_m1
Mouse	TROLL-3 (E130307A14Rik)	Mm04237550_m1
Mouse	TROLL-4 (2610307P16Rik)	Mm01129970_m1
Mouse	TROLL-5 (AC122821.1)	Mm00467926_m1
Mouse	TROLL-6 (2410006H16Rik)	Mm01331817_g1
Mouse	TROLL-7 (Malat1)	Mm01227912_s1
Mouse	TROLL-8 (AC157572.1)	Mm01731578_gH
Mouse	TROLL-9 (4930481B07)	Mm01257798_g1
Mouse	GAPDH	Mm99999915_g1
Human	TROLL-1 (RP11-98G7.1)	Hs00418864_m1
Human	TROLL-2 (RPSAP52)	Hs03677485_m1
Human	TROLL-3 (TRAF3IP2-AS1)	Hs04274045_m1
Human	TROLL-4 (NR_015410)	Hs01371948_m1
Human	TROLL-5 (LINC00514)	Hs04273767_g1
Human	TROLL-6 (RP11-138I1.3)	Hs00415106_m1
Human	TROLL-7 (MALAT1)	Hs00273907_s1
Human	TROLL-8 (AL161668.12)	Hs00922963_s1
Human	TROLL-9 (RP11-126K1.6)	Hs04937869_g1
Human	TAp63	Hs00186613_m1
Human	IFIT1	Hs03027069_s1
Human	ITG3BP	Hs00183924_m1
Human	KCTD7	Hs00399233_m1
Human	MAD2L2	Hs01057448_m1
Human	NCOA5	Hs01001913_m1
Human	TERB2	Hs01014975_m1
Human	WDR26	Hs01553459_m1
Human	POLR2A	Hs00172187_m1

Supplementary Table 2 Sequences of siRNA pools.

Species	Gene Name	Sequence (Sense, 5'-3')
Human	TROLL-1 (RP11-98G7.1)	GUAACUGAAAGUUACUUAA
		UCAAUAAUCUCCAUGUUAG
		CUCACAAGAAGGCUUCAAU
Human	TROLL-2 (RPSAP52)	CACUGUUACUCAGCCUGAA
		GAAGGAAUUUCAGGGUAAA
		CCUGGAACCUUCCUAACCA
Human	TROLL-3 (TRAF3IP2-AS1)	GAGCAUCAUUUAGAAGAGG
		GCUGGUCACAAACUUCCUG
		GCTATGCAGGATTGGCAGG
Human	TROLL-4 (NR_015410)	GAAGUACCCUCAGGUGACU
		CAUCUAGAAAGCAUCAUGA
		GACUUGAGGGUCAACUUUA
Human	TROLL-5 (LINC00514)	CACACUCUGGGUCCCAACA
		CCUCUACCCUCCUGUGACA
		CUGUGUCUGCCCUCAAACA
Human	TROLL-6 (RP11-138I1.3)	CAUAUCAAUUUAAACUUCA
		GGACUACAAAUCUUAAUUA
		GUUGUAAUCUCCAUUUCAG
Human	TROLL-7 (MALAT1)	GAAUUCCGGUGAUGCGAGU
		CUAACGAUUUGGUGGUGAA
		GUAAAGCCCUGAACUAUCA
Human	TROLL-8 (AL161668.12)	CAUCCAUAAAGAAGGCAUA
		CCACUUAUUGGCCCUCAUU
		GACUUGUUCUGUCGCUUCU
Human	TROLL-9 (RP11-126K1.6)	AGUUUAGUCUACGGUCCUC
		UGGAUGAUAAAAUAACUAG
		CUUUUUCUGCUCUCCUCCC

Supplementary Table 3 List of primers for ChIP assay.

Promoter	Binding Site	Primers (5'-3')
TROLL-1(RP11-98G7.1)	TAp63 BS (-2238 to -2213)	Forward: CAGAGTGCACTTTCACATGC
		Reverse: TGCACTGCCTTGTCCATGTT
	non-specific (-914 to -936)	Forward: GTAGTGACACAGGCGAGGAC
		Reverse: CACGTGCCCCATAAAAGCTG
TROLL-2(RPSAP52)	TAp63 BS (-4269 to -4240)	Forward: TGCAAGGGTTCAAAGAGGAAACT
		Reverse: ATGAGTTGCCAAATGCATAGCC
	non-specific (-297 to -273)	Forward: TGAAAAGCAAACCACATTTCGAG
		Reverse: ACAGATCTAGGAAGCAAAGTGC
TROLL-3(TRAF3IP2-AS1)	TAp63 BS (-3825 to -3800)	Forward: TGTCGCCCAGGCTGGTCTCC
		Reverse: TCTTAAGTTGGTGAAATTTG
	non-specific (-741 to -716)	Forward: GGGCTGGCCATGTAGTAGTC
		Reverse: CTCCTCGAGGTGCCTCTGT
TROLL-4(NR_015410)	TAp63 BS (-4919 to -4898)	Forward: ACTCAGTTTGGTATCCAGGG
		Reverse: TGTTTGTCTCTTCCAGGTGG
	non-specific (-1101 to -1079)	Forward: TGCTATGGCCCGAAGTTGGG
		Reverse: CAGGAACAGCTCCCGACC
TROLL-5(LINC00514)	TAp63 BS (-2320 to -2297)	Forward: CTGTGTGAATCCAGCACCC
		Reverse: AGCTGGGACGGTCCATTC
	non-specific (-428 to -406)	Forward: GAGAATTCAGACAGTGAGG
		Reverse: TGCGGAAGCAAGACAGTGC
TROLL-6(RP11-138I1.3)	TAp63 BS (-2287 to -2264)	Forward: GCTGGCCTCCAGGATCGC
		Reverse: CCTGTAAACAGATGGCAAGC
	non-specific (-683 to -658)	Forward: AGGTCTTATGATTGTCTTCCC
		Reverse: CCTTTCAGCCTATGAACATGC

TROLL-7(MALAT1)	TAp63 BS (-3874 to -3851)	Forward: GCACTCTAGCCTGGGTGAC
		Reverse: TCTTCATGGCACCTGGATTCC
	non-specific (-695 to -674)	Forward: ACAGCGTCACTAATCTCTCC
		Reverse: CCTAAAATGGAGAGTTCCAGG
TROLL-8 (AL161668.12)	TAp63 BS (-2034 to -2013)	Forward: CTGCCTGATCTCCATGTAGC
		Reverse: CACTGTAGTTGAGAAAATGAGC
	non-specific (-3379 to -3359)	Forward: TGAAGAGAATGAGAAAATGTGG
		Reverse: CAAAGTGCTAGGATTACAGGC
TROLL-9(RP11-126K1.6)	TAp63 BS (-3278 to -3254)	Forward: CTGAAAGTGGCTCAGGGTTG
		Reverse: GAGCGGATCCTGAAACGGT
	non-specific (-330 to -309)	Forward: TCCATTCTATCTGCCCCACCT
		Reverse: TTGTTTTTGAGGGGGAGGAGG

Supplementary Table 4 List of human cancer tissue microarrays (TMAs).

TMA Name	TMA ID and Source	Pathology Diagnosis
Breast cancer progression TMA – Biomax	BR480a (US Biomax)	Normal breast tissue (n = 2)<br>Lobular hyperplasia (n = 10)<br>Ductal carcinoma in situ (n = 11)<br>Invasive breast cancer (n = 22)
Invasive breast cancer TMA – Biomax	BR20837a (US Biomax)	Grade 1 (n = 4)<br>Grade 2 (n = 34)<br>Grade 3 (n = 39)
Invasive breast cancer TMA – Dundee	Breast TMA (26), Tayside Tissue Bank	Grade 1 (n = 18)<br>Grade 2 (n = 58)<br>Grade 3 (n = 79)
Invasive breast cancer TMA – Moffitt	TMA-5, Moffitt Cancer Center	Triple negative breast cancer (TNBC) (n = 43)<br>Non-TNBC (n = 25)
Colon cancer TMA	CO961 (US Biomax)	Normal colon tissue (n = 1)<br>Polyp (n = 5)<br>Adenoma (n = 6)<br>Adenocarcinoma (n = 61)
Lung adenocarcinoma TMA	BC04002a (US Biomax)	Normal lung tissue (n = 2)<br>Benign lesion (n = 9)<br>Adenocarcinoma (LUAD) (n = 16)
Lung squamous cell carcinoma TMA	BC04002a (US Biomax)	Normal lung tissue (n = 2)<br>Benign lesion (n = 9)<br>Squamous cell carcinoma (LUSC) (n = 18)<br>Metastatic LUSC (n = 10)
Melanoma TMA – Biomax	ME1004 (US Biomax)	Benign nevus (n = 16)<br>Stage 1 (n = 3)<br>Stage 2 (n = 30)<br>Stage 3 (n = 12)<br>Stage 4 (n = 1)<br>Unknown stage (n = 31)
Melanoma TMA – Moffitt	TMA-4, Moffitt Cancer Center	Control case (n = 6)<br>Stage 3 (n = 39)<br>Stage 4 (n = 61)
Ovarian cancer – Biomax	OV1005b (US Biomax)	Normal ovary (n = 6)<br>Serous adenocarcinoma (n = 32)<br>Non-Serous adenocarcinoma (n = 13)

Supplementary Table 5 List of List of primers for CLIP-qPCR assay.

Name	Primers (5'-3')
TROLL-2 segment 1 (5 to 127)	Forward: CCCATTTAGAGAATTCGGGAAG
	Reverse: AGTTAAGGCAGAGTTCACAAAG
TROLL-2 segment 2 (116 to 208)	Forward: TCTGCCTTAACTCAAAGATTCC
	Reverse: CATCCTCCTCCTTCATTTGC
TROLL-2 segment 3 (189 to 317)	Forward: GCAAATGAAGGAGGAGGATG
	Reverse: TCAGATTTATGCTGTAGATGCC
TROLL-2 segment 4 (303 to 403)	Forward: ACAGCATAAATCTGAAGAGGAC
	Reverse: CCTGGAGGATATGACACTGAC
TROLL-2 segment 5 (382 to 540)	Forward: TGTCAGTGTCATATCCTCCAG
	Reverse: GTCAGAAACCACAAGAAGCC
TROLL-2 segment 6 (489 to 636)	Forward: TAACCAGATCCAGGCAGCCTAC
	Reverse: TGTCCAATGGCACAGAGGAG
TROLL-2 segment 7 (627 to 738)	Forward: CCATTGGACATTGCCATCAC
	Reverse: TGTTGCAGGAAATGGTGC
TROLL-2 segment 8 (722 to 864)	Forward: CACCATTTCCTGCAACACC
	Reverse: GCTGAAACAGTCCATTTACCC
TROLL-2 segment 9 (807 to 944)	Forward: CTGCTGCTGAAAAGGCTGTGAC
	Reverse: ACTGCTGAATAGGCACAGAGGG
TROLL-2 segment 10 (929 to 1058)	Forward: GTGCCTATTCAGCAGTTTCTAC
	Reverse: TGCAAGAACAGCTTAAGACC
TROLL-2 segment 11 (1038 to 1108)	Forward: TGGTCTTAAGCTGTTCTTGCAC
	Reverse: ACTGATGTTTATTTTCTGTCAACCT
TROLL-3 segment 1 (7 to 117)	Forward: GCTTGCGGGAGGGGGGC
	Reverse: CTGCTGGATGTGAAATGGCG
TROLL-3 segment 2 (96 to 195)	Forward: ACCGCCATTTCACATCCAG
	Reverse: TGGGCAATATAGTGAGACCTC
TROLL-3 segment 3 (187 to 292)	Forward: TATTGCCCAAGCTGGTCTC
	Reverse: ATAATTTCGCAGGGCATGG
TROLL-3 segment 4 (255 to 360)	Forward: AGATAACAGGTATGAGCCACC
	Reverse: GCAGTGAGTTGAGATTGGG
TROLL-3 segment 5 (342 to 489)	Forward: CCCAATCTCAACTCACTGC
	Reverse: GGGCAATATAGTGAAACCCTG
TROLL-3 segment 6 (472 to 621)	Forward: GGTTTCACTATATTGCCCAGG

	Reverse: AAGGAAAGAAAAGGGATGACAC
TROLL-3 segment 7 (603 to 749)	Forward: TCATCCCTTTTCTTTCCTTCAC
	Reverse: AATGCCTCCAGATATTTTGCC
TROLL-3 segment 8 (727 to 837)	Forward: TTGGCAAAATATCTGGAGGC
	Reverse: TTCAACACCTGCACTATTGAC
TROLL-3 segment 9 (805 to 943)	Forward: CAGGTCCAAAGTGTCAATAGTG
	Reverse: AAATGCCTCTGAAAGGGAATG
TROLL-3 segment 10 (884 to 1029)	Forward: AGTCTCTTGCCTTTATGAAACC
	Reverse: GCAGAAACCTGCATATAAAGCC
TROLL-3 segment 11 (972 to 1071)	Forward: TGGAGTCAATTGCATCAACC
	Reverse: TATGTGCCTACCTTGTACCC
TROLL-3 segment 12 (1051 to 1185)	Forward: TGGGTACAAGGTAGGCACAT
	Reverse: GGAAATTCATCTCTCACCACATGA
TROLL-3 segment 13 (1168 to 1284)	Forward: GGTGAGAGATGAATTTCCTGAACA
	Reverse: TGCTAACTTATGGAAGTGGAAGTT
TROLL-3 segment 14 (1261 to 1412)	Forward: ACTTCCACTTCCATAAGTTAGCAG
	Reverse: AATGAGGGTCCTGGAAAGCTC
TROLL-3 segment 15 (1391 to 1496)	Forward: TGAGCTTTCCAGGACCCTCATT
	Reverse: TCTATGTGTGTTTGGTCTCCTCC
TROLL-3 segment 16 (1475 to 1603)	Forward: GAGGAGACCAAACACACATAGAGT
	Reverse: TTTGGGGCCAGGGGAAACTAC
TROLL-3 segment 17 (1592 to 1666)	Forward: CCTGGCCCCAAAGTTTATTGC
	Reverse: AGCTTTTTAGTGGGAGTTTTTCTGG
TROLL-3 segment 18 (1637 to 1747)	Forward: ACATTCCAGAAAAACTCCCAC
	Reverse: GGGCTCAAGCAATAATCTCAC
TROLL-3 segment 19 (1742 to 1841)	Forward: GAGCCCAGAAGTTTGAGACC
	Reverse: GGGACTACAGGCATACATCAC
TROLL-3 segment 20 (1819 to 1966)	Forward: TGGTGATGTATGCCTGTAGTC
	Reverse: TGTGTGTTTTTTGAGACAGGG
TROLL-3 segment 21 (1906 to 2045)	Forward: ATGATCACCACTGCACTCC
	Reverse: CTTTTCTGGCCTCTGGAATG
TROLL-3 segment 22 (2025 to 2174)	Forward: TCATTCCAGAGGCCAGAAAAG
	Reverse: TTCTTTATTGTCCTCCATGTTTTCC
