# Supplementary material for: Pan-cancer analysis reveals TAp63-regulated oncogenic lncRNAs that promote cancer progression through AKT activation
Source: Nat Commun. 2020 Oct 14;11:5156. doi: 10.1038/s41467-020-18973-w (PMC7561725; doi:10.1038/s41467-020-18973-w)
Supplement: Supplementary file 3 — Description of Additional Supplementary Files [file 41467_2020_18973_MOESM3_ESM.pdf]

Description of Additional Supplementary Files

File Name: Supplementary Data 1
Description: List of peptides interacting with WDR26 and its mutants
