# Supplementary material for: Pan-cancer analysis reveals TAp63-regulated oncogenic lncRNAs that promote cancer progression through AKT activation
Source: Nat Commun. 2020 Oct 14;11:5156. doi: 10.1038/s41467-020-18973-w (PMC7561725; doi:10.1038/s41467-020-18973-w)
Supplement: Supplementary file 5 — Reporting Summary [file 41467_2020_18973_MOESM5_ESM.pdf]

nature research

Corresponding author(s): Elsa R. Flores

Last updated by author(s): Sep 4, 2020

Reporting Summary

Nature Research wishes to improve the reproducibility of the work that we publish. This form provides structure for consistency and transparency in reporting. For further information on Nature Research policies, see our Editorial Policies and the Editorial Policy Checklist.

Statistics

For all statistical analyses, confirm that the following items are present in the figure legend, table legend, main text, or Methods section.

n/a	Confirmed	
☐	☒	The exact sample size (n) for each experimental group/condition, given as a discrete number and unit of measurement
☐	☒	A statement on whether measurements were taken from distinct samples or whether the same sample was measured repeatedly
☐	☒	The statistical test(s) used AND whether they are one- or two-sided<br>*Only common tests should be described solely by name; describe more complex techniques in the Methods section.*
☒	☐	A description of all covariates tested
☒	☐	A description of any assumptions or corrections, such as tests of normality and adjustment for multiple comparisons
☐	☒	A full description of the statistical parameters including central tendency (e.g. means) or other basic estimates (e.g. regression coefficient) AND variation (e.g. standard deviation) or associated estimates of uncertainty (e.g. confidence intervals)
☐	☒	For null hypothesis testing, the test statistic (e.g. F, t, r) with confidence intervals, effect sizes, degrees of freedom and P value noted<br>*Give P values as exact values whenever suitable.*
☒	☐	For Bayesian analysis, information on the choice of priors and Markov chain Monte Carlo settings
☒	☐	For hierarchical and complex designs, identification of the appropriate level for tests and full reporting of outcomes
☒	☐	Estimates of effect sizes (e.g. Cohen's d, Pearson's r), indicating how they were calculated

Our web collection on statistics for biologists contains articles on many of the points above.

Software and code

Policy information about availability of computer code

Data collection	Microsoft Excel v16.16.5
Data analysis	GraphPad Prism v7.0d, Oncotopix Visiopharm v2018.4, Circos v0.69, TopHat v2.0.12, R v3.6.1

For manuscripts utilizing custom algorithms or software that are central to the research but not yet described in published literature, software must be made available to editors and reviewers. We strongly encourage code deposition in a community repository (e.g. GitHub). See the Nature Research guidelines for submitting code & software for further information.

Data

Policy information about availability of data

All manuscripts must include a data availability statement. This statement should provide the following information, where applicable:

- Accession codes, unique identifiers, or web links for publicly available datasets
- A list of figures that have associated raw data
- A description of any restrictions on data availability

Public available data used in this paper were obtained from UCSC (http://genome.ucsc.edu/cgibin/hgGateway) and The Cancer Genome Atlas (TCGA) breast cancer and melanoma datasets (http://www.cancer.gov/about-nci/organization/ccg/research/structural-genomics/tcga).
The source data underlying Figs. 1a-h, 2a-d, 2f, 2h, 3b-e, 4b-f, 5a, 5c, 5e, 6a-e and Supplementary Figs. 1a-i, 2a-m, 2o, 2q, 3a-k, 4a-h, 5a-m', 5o', 5q', 5s', 5u', 5x'-a", 5c", 5e", 6a-6l" are provided as Source Data File. All the other data (imaging) supporting the findings of this study are available from the corresponding author upon reasonable request.

Field-specific reporting

Please select the one below that is the best fit for your research. If you are not sure, read the appropriate sections before making your selection.

☒ Life sciences ☐ Behavioural & social sciences ☐ Ecological, evolutionary & environmental sciences

For a reference copy of the document with all sections, see nature.com/documents/nr-reporting-summary-flat.pdf

Life sciences study design

All studies must disclose on these points even when the disclosure is negative.

Sample size	For all the in vitro experiments, a minimum of three biological replicates were used per sample. For all the in vivo experiment, a minimum of five mice were used per each group. No statistical method was used to determine the sample/group size.
Data exclusions	None of the replicates was excluded from any of the presented data.
Replication	Experiments were successfully performed at least three times. All replication attempts were successful.
Randomization	Mice were randomly allocated into different experimental groups before being injected with the cell lines of interest. For experiments involving cellular and biological studies, three independent experiments have been performed, allocating randomly cells in experimental groups.
Blinding	Tumour volume assessment and the quantification of lung nodules were blinded. The IHC and ISH signals and the proportion of positive tissue were measured blindly. For most of the other experiments, the results were quantified and appropriate statistical tests were performed to evaluate difference and statistical significance.

Reporting for specific materials, systems and methods

We require information from authors about some types of materials, experimental systems and methods used in many studies. Here, indicate whether each material, system or method listed is relevant to your study. If you are not sure if a list item applies to your research, read the appropriate section before selecting a response.

Materials & experimental systems

n/a	Involved in the study	
☐	☒	Antibodies
☐	☒	Eukaryotic cell lines
☒	☐	Palaeontology and archaeology
☐	☒	Animals and other organisms
☒	☐	Human research participants
☒	☐	Clinical data
☒	☐	Dual use research of concern

Methods

n/a	Involved in the study	
☒	☐	ChIP-seq
☒	☐	Flow cytometry
☒	☐	MRI-based neuroimaging

Antibodies

Antibodies used

Chromatin Immunoprecipitation:
TAp63 (sc-8608, Santa Cruz)
Normal mouse IgG (sc-2025, Santa Cruz)
Normal rabbit IgG (sc-2027, Santa Cruz)

Immunoprecipitation:
AKT (9272S, Cell Signaling)
NOLC1 (ab184550, Abcam)
Normal rabbit IgG (sc-2027, Santa Cruz)
WDR26 (ab203345, Abcam)

Immunohistochemistry:
NCOA5 (ab70831, Abcam)
WDR26 (ab203345, Abcam)
pAKT (S473) (4060S, Cell Signaling)
TAp63 (618902, Biolegend)

Western Blot:
FLAG (A8592, Sigma)
WDR26 (ab85961, Abcam)

	H3 (ab1791, Abcam)<br>HSP90 (ab13495)<br>pAKT (S473) (4060S, Cell Signaling)<br>AKT (ab8805, Abcam)<br>Actin (A5441, Sigma)<br>NOLC1 (sc-374033, Santa Cruz)
Validation	Antibody: Actin (A5441, Sigma)<br>Species reactivity: multiple species including human and mouse<br>Applications validated by manufacturer: IHC, IF, WB<br><br>Antibody: AKT (9272S, Cell Signaling)<br>Species reactivity: multiple species including human and mouse<br>Applications validated by manufacturer: IP, WB<br><br>Antibody: AKT (ab8805, Abcam)<br>Species reactivity: mouse and human<br>Applications validated by manufacturer: IHC, WB<br><br>Antibody: pAKT (S473) (4060S, Cell Signaling)<br>Species reactivity: multiple species including human and mouse<br>Applications validated by manufacturer: IHC, IP, WB<br><br>Antibody: FLAG (A8592, Sigma)<br>Species reactivity: all<br>Applications validated by manufacturer: WB<br><br>Antibody: H3 (ab1791, Abcam)<br>Species reactivity: multiple species including human and mouse<br>Applications validated by manufacturer: WB<br><br>Antibody: HSP90 (ab13495)<br>Species reactivity: mouse and human<br>Applications validated by manufacturer: WB<br><br>Antibody: NCOA5 (ab70831, Abcam)<br>Species reactivity: human<br>Applications validated by manufacturer: IHC, IP, WB<br><br>Antibody: NOLC1 (ab184550, Abcam)<br>Species reactivity: mouse and human<br>Applications validated by manufacturer: IP, WB<br><br>Antibody: NOLC1 (sc-374033, Santa Cruz)<br>Species reactivity: human<br>Applications validated by manufacturer: IP, WB<br><br>Antibody: Normal mouse IgG (sc-2025, Santa Cruz)<br>Validated by manufacturer as a negative control for IP<br><br>Antibody: Normal rabbit IgG (sc-2027, Santa Cruz)<br>Validated by manufacturer as a negative control for IP<br><br>Antibody: TAp63 (sc-8608, Santa Cruz)<br>Species reactivity: mouse and human<br>Applications validated by manufacturer: IP, WB<br><br>Antibody: TAp63 (618902, Biolegend)<br>Species reactivity: human<br>Applications validated by manufacturer: IHC, WB<br><br>Antibody: WDR26 (ab85961, Abcam)<br>Species reactivity: human<br>Applications validated by manufacturer: WB<br><br>Antibody: WDR26 (ab203345, Abcam)<br>Species reactivity: mouse and human<br>Applications validated by manufacturer: IHC, IP

Eukaryotic cell lines

Policy information about cell lines

Cell line source(s)	The MCF10A progression model cell lines (MCF10A, AT1, DCIS, and CA1D) were obtained from the Karmanos Cancer Center (Detroit, USA). MDA MB-231, A375, H1299, H358, and Malme-3M cells were obtained from ATCC.

Authentication	All the cell lines used were authenticated via STR loci profiling at the Molecular Genomics Core (Moffitt Cancer Center).
Mycoplasma contamination	All the cell lines used were mycoplasma negative.
Commonly misidentified lines (See ICLAC register)	No commonly misidentified cell lines were used.

Animals and other organisms

Policy information about studies involving animals; ARRIVE guidelines recommended for reporting animal research

Laboratory animals	All the mice used in this study were six-to-eight week old female athymic nu/nu mice.
Wild animals	The study did not involve wild animals.
Field-collected samples	The study did not involve field-collected samples.
Ethics oversight	All procedures were approved by the IACUC at the H. Lee Moffitt Cancer Center & Research Institute.

Note that full information on the approval of the study protocol must also be provided in the manuscript.
